# Incidence and Mortality of COVID-19-Associated Invasive Fungal Infections Among Critically Ill Intubated Patients: A Multicenter Retrospective Cohort Analysis

**DOI:** 10.1093/ofid/ofae108

**Published:** 2024-04-02

**Authors:** Julio C Zuniga-Moya, Benjamin Papadopoulos, Armaghan-E-Rehman Mansoor, Patrick B Mazi, Adriana M Rauseo, Andrej Spec

**Affiliations:** St Louis School of Medicine, Washington University, St Louis, Missouri, USA; St Louis School of Medicine, Washington University, St Louis, Missouri, USA; St Louis School of Medicine, Washington University, St Louis, Missouri, USA; St Louis School of Medicine, Washington University, St Louis, Missouri, USA; St Louis School of Medicine, Washington University, St Louis, Missouri, USA; St Louis School of Medicine, Washington University, St Louis, Missouri, USA

**Keywords:** invasive fungal infections, SARS-CoV-2

## Abstract

**Background:**

An association between coronavirus disease 2019 (COVID-19)–associated invasive fungal infections (CAIFIs) and high mortality among intubated patients has been suggested in previous research. However, some of the current evidence was derived from small case series and multicenter studies conducted during different waves of the COVID-19 pandemic. We examined the incidence of CAIFIs and their associated mortality using a large, multicenter COVID-19 database built throughout the pandemic.

**Methods:**

We conducted a retrospective analysis of the National COVID Cohort Collaborative (N3C) database collected from 76 medical centers in the United States between January 2020 and August 2022. Patients were 18 years or older and intubated after severe acute respiratory syndrome coronavirus 2 infection. The primary outcomes were incidence and all-cause mortality at 90 days. To assess all-cause mortality, we fitted Cox proportional hazard models after adjusting for confounders via inverse probability weighting.

**Results:**

Out of the 4 916 229 patients with COVID-19 diagnosed during the study period, 68 383 (1.4%) met our cohort definition. The overall incidence of CAIFI was 2.80% (n = 1934/68 383). *Aspergillus* (48.2%; n = 933/1934) and *Candida* (41.0%; n = 793/1934) were the most common causative organisms. The incidence of CAIFIs associated with *Aspergillus* among patients who underwent BAL was 6.2% (n = 83/1328). Following inverse probability weighting, CAIFIs caused by *Aspergillus* (hazard ratio [HR], 2.0; 95% CI, 1.8–2.2) and *Candida* (HR, 1.7; 95% CI, 1.5–1.9) were associated with increased all-cause mortality. Systemic antifungals reduced mortality in 17% of patients with CAIFI with *Aspergillus* and 24% of patients with CAIFI with *Candida*.

**Conclusions:**

The incidence of CAIFI was modest but associated with higher 90-day all-cause mortality among intubated patients. Systemic antifungals modified mortality.

Invasive fungal infections have increased significantly since the start of the coronavirus disease 2019 (COVID-19) pandemic [1]. Further, among critically ill COVID-19 patients, invasive fungal infections (IFIs) have emerged as the second most common secondary infection [2, 3]. However, the reported incidence of COVID-19-associated invasive fungal infections (CAIFIs) has varied globally during different COVID-19 waves and with different cohort designs and diagnostic criteria [4–8]. CAIFI incidence ranges from 5% to 30% in critically ill patients [9–12]. Similar heterogeneity is seen in mortality rates [13]. The available evidence indicates a notable increase in mortality related to fungal infections during the pandemic, particularly in cases of CAIFI [14]. CAIFI associated with *Aspergillus* spp. has been shown to double 90-day mortality [9]. *Candida* spp. and mucormycosis have been reported to have 30%–80% and 50% crude mortality [15–17].

Data from early studies were crucial for clinicians caring for patients with COVID-19, especially early in the pandemic. However, many studies able to collect and disseminate this critical knowledge were single-center studies [2, 3, 7, 8, 18], were conducted only during specific pandemic waves [3, 10, 12, 19], or reported on limited cases [4, 5].

Medical mycology conventionally restricts the application of causal inference to randomized controlled trials (RCTs). However, the astonishingly few RCTs in the field limits progress on improving patient-centered outcomes. Despite its intrinsic limitations, there is an abundance of observational data primed for analysis with modern statistical approaches. We sought to assess the incidence and mortality associated with CAIFI among critically ill patients with COVID-19 using the largest multicenter database of patients with COVID-19 across the entire pandemic. By employing a rigorous causal inference framework, our approach aims to build upon the valuable insights from prior observational studies, enhancing our ability to provide a more nuanced perspective of the latter association.

## METHODS

### Data Source

**Figure 1.**
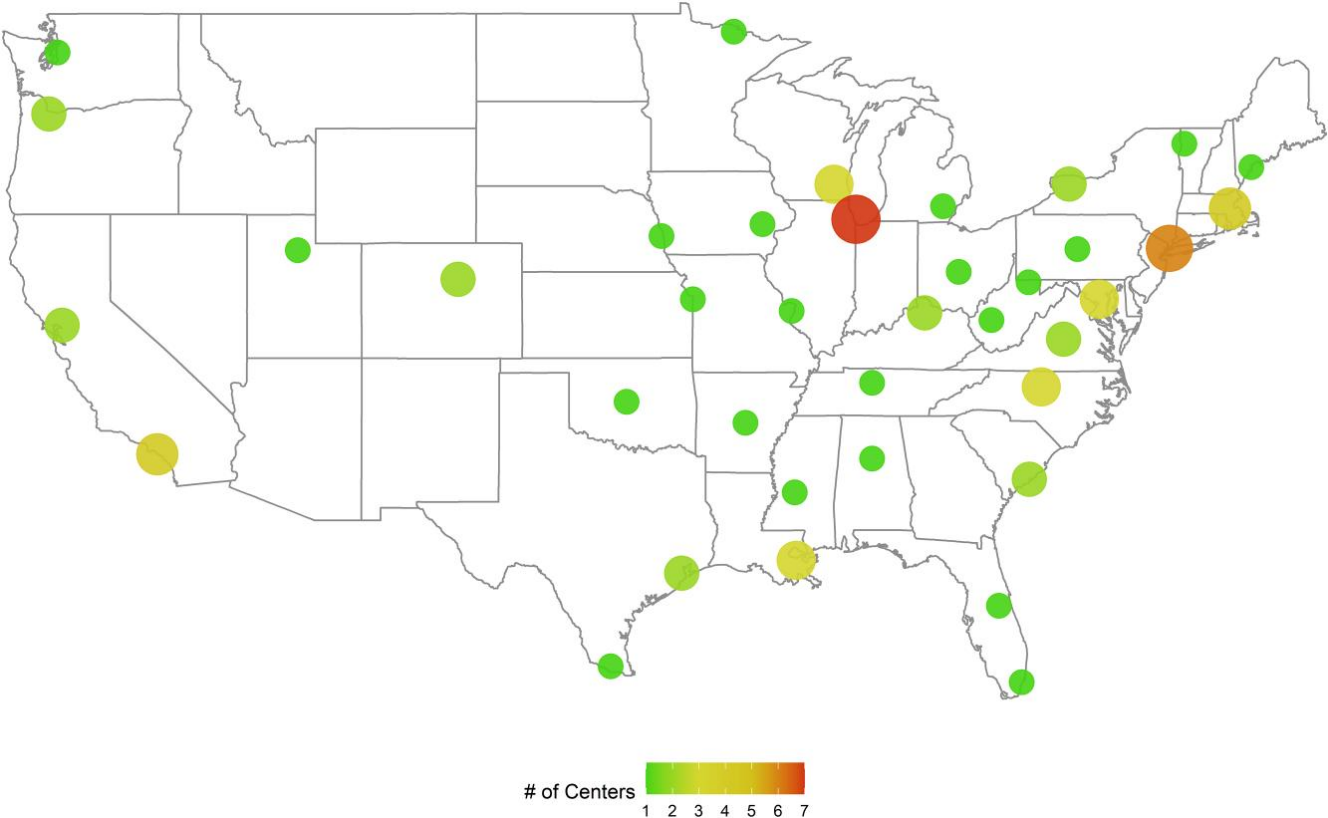
Distribution of 76 centers within the N3C collaboration in the United States.

We utilized data from the National COVID Cohort Collaborative (N3C) [20]. The N3C is an initiative of the National Institutes of Health utilizing electronic health record data since 2018 among 76 US health care institutions. At the time of data sampling (January 1, 2020, to August 19, 2022), there were 4 916 229 patients with COVID-19 and 3 uninfected controls (Figure 1). For every COVID-19-infected patient hospitalized, 3 control patients were identified who were also hospitalized but with a negative severe acute respiratory syndrome coronavirus 2 (SARS-CoV-2) test. We received authorization for data use (RP-287159).

### Study Population and Study Design

We conducted a retrospective cohort study of adults age 18 years or older with positive SARS-CoV-2 polymerase chain reaction (PCR) or antigen testing who were intubated following their COVID-19 diagnosis. Positive PCR testing was used to define patients with influenza and respiratory syncytial virus (RSV). Intubation associated with COVID-19 was defined as occurring within 30 days following positive SARS-CoV-2 testing. The intubation date was the index date for survival analysis. Patients who died within 24 hours of intubation were excluded due to insufficient time to complete a CAIFI diagnostic workup.

Comorbidities and medications associated with COVID-19 treatment (Supplementary Table 1) were defined using 3 classifications: International Classification of Diseases, Ninth and Tenth Editions (ICD-10 and ICD-9), and Systematized Nomenclature of Medicine - Clinical Terms (SNOMED-CT) if they were present during the index hospitalization or the preceding 12 months (Supplementary Table 2). Clinical investigators (A.S., P.M., A.E.M., and A.R.) reviewed each concept and code to avoid misclassification bias (Supplementary Table 2).

### Patient Consent

No informed consent was obtained from patients because of the nature of the data set.

### Exposure

CAIFIs were defined as invasive infections associated with the following organisms: *Aspergillus*, Mucorales, *Candida*, *Cryptococcus*, *Blastomyces*, *Histoplasma*, and *Coccidioides*. Diagnosis required:

1. a positive laboratory test for any CAIFI defined previously in guidelines (Supplementary Table 3) [21, 22]
  or
2. a clinical diagnosis via documentation of specific ICD-9, ICD-10, or SNOMED-CT codes (Supplementary Table 4).

The first positive test/diagnosis date occurring after intubation defined the time of CAIFI diagnosis.

### Outcome

The primary outcomes were CAIFI incidence and 90-day all-cause mortality. The secondary outcome was 30-day all-cause mortality.

### Statistical Analysis

The IFI incidence was examined in both COVID-19-positive and COVID-19-negative patients, utilizing proportions as a means of representation. Sensitivity analyses were conducted to explore the incidence of IFIs, specifically among patients intubated for a duration exceeding 96 hours. Patients intubated for a shorter time period were at much lower risk for developing complications.

Fungal pathogens were analyzed individually. The control population was comprised of patients with COVID-19 but without an IFI. We adjusted for confounding in survival models [23, 24]. Elixhauser comorbidities, historical IFI risk factors, and clinically plausible risk factors (chosen a priori) were used for adjustment (Supplementary Tables 1–2). We examined any differences within the covariates after weighting (Supplementary Figures 1–7) [25].

We fitted Cox proportional hazard models to examine 30-day and 90-day all-cause mortality for each pathogen. A stratified analysis was conducted for pandemic waves (Wuhan-HU-1, Delta, and Omicron) [26]. Time-dependent exposures were used to reduce immortal time bias [27].

A sensitivity analysis compared ICD/SNOMED-CT codes and lab testing for 90-day all-cause mortality, while effect modification by treatment status was analyzed. Cox models assessed each CAIFI as an exposure and 90-day mortality as outcomes. We defined treatment for IFIs if the data in the database adhered to relevant guidelines [21, 28–31]. A more detailed description of the statistical analysis can be found in the Supplementary Methods.

Data management and preparation for analysis were performed using Spark SQL (Apache-spark.org; version 3.0.2), while the descriptive analysis and survival analysis were performed using SparkR (r-project.org; version 4.2.1) within the N3C database.

## RESULTS

**Figure 2.**
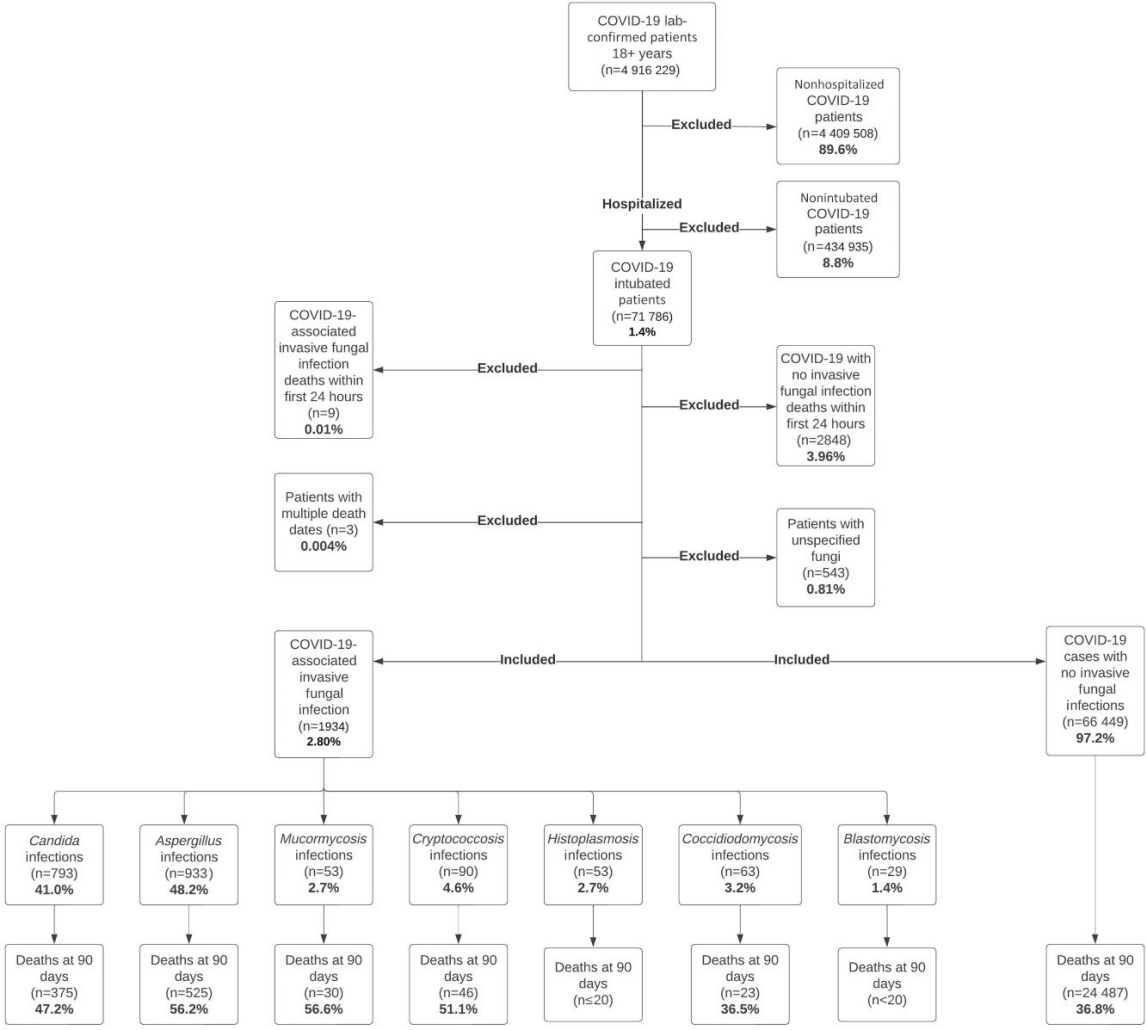
Inclusion/exclusion criteria for the composition of the analysis cohort. Abbreviation: COVID-19, coronavirus disease 2019.

A total of 68 383 patients met inclusion criteria, and 1934 (2.80%) were diagnosed with CAIFI (Figure 2). CAIFI diagnosis was identified solely by ICD/SNOMED-CT codes in 82.4% (n = 1595) of cases, solely by laboratory testing in 7.9% (n = 154), and by both methods in 9.7% (n = 185). *Aspergillus* was the most common pathogen (1.3%; n = 933/68 383), followed by *Candida* (1.1%; n = 793/68 383), *Cryptococcus* (0.1%; n = 90/68 383), *Coccidioides* (0.09%; n = 63/68 383), Mucormycetes (0.08%; n = 53/68 383), *Histoplasma* (0.07%; n = 53/68 383), and *Blastomyces* (0.04%; n = 29/68 383) (Figure 2). CAIFI associated with *Candida* and *Aspergillus* was the most common coinfection in our cohort (0.04%; n = 32/68 383). Patient demographic and comorbidity data are presented in Table 1.

**Table 1.** Baseline Characteristics, Comorbidities, and Associated Medications Among Patients With COVID-19-Associated Invasive Fungal Infections Within Multiple Centers in the United States (2020–2022)

	*Aspergillus* Infections n = 933 (%)	*Candida* Infections n = 793 (%)	Other Invasive Fungal Infections^b^ n = 282 (%)	No Invasive Fungal Infections n = 66 449 (%)	Total Cohort^a^ n = 68 383 (%)	*P* Value^c^
Demographics						
Age, mean (SD), y	61.0 (13.6)	58.7 (14.9)	57.2 (13.6)	62.3 (15.5)	62.0 (15.5)	<.0001
Male sex	564 (60.4)	498 (62.7)	203 (71.9)	36 405 (54.7)	37 623 (55.0)	<.0001
Race						<.0001
White	622 (66.6)	419 (52.8)	173 (61.3)	38 805 (58.3)	39 974 (58.4)	
Black	135 (14.4)	183 (23.1)	51 (18.1)	11 890 (17.8)	12 251 (17.9)	
Asian	34 (3.6)	29 (3.7)	<20	1342 (2.3)	1411 (2.1)	
Others/not reported	142 (15.2)	162 (20.4)	51 (18.1)	14 379 (21.6)	14 747 (21.6)	
Preexisting medical conditions						
Diabetes	527 (56.5)	427 (53.8)	166 (58.9)	30 476 (45.9)	31 545 (46.1)	<.0001
Hypertension	767 (82.2)	654 (82.5)	233 (82.6)	47 082 (70.9)	48 681 (71.2)	<.0001
Congestive heart failure	348 (37.3)	320 (40.4)	111 (39.4)	22 303 (33.6)	23 052 (33.7)	<.0001
Obesity	430 (46.1)	351 (44.3)	123 (43.6)	27 079 (40.8)	27 946 (40.9)	<.0001
Cardiac arrhythmia	251 (26.9)	188 (23.7)	71 (25.2)	14 745 (22.2)	15 237 (22.3)	.001
Renal failure	257 (27.5)	174 (21.9)	99 (35.1)	13 006 (19.6)	13 516 (19.8)	<.0001
Coagulopathy	551 (59.1)	527 (66.5)	175 (62.1)	22 686 (34.1)	23 885 (34.9)	<.0001
Valvular disease	255 (27.3)	271 (34.2)	89 (31.6)	14 684 (22.1)	15 276 (22.3)	<.0001
Metastatic cancer	39 (4.2)	29 (3.7)	<20	2232 (3.4)	2311 (3.4)	.09
HIV	<20	<20	<20	432 (.7)	463 (.7)	<.0001
Leukemia	46 (4.9)	23 (2.9)	<20	1106 (1.7)	1181 (1.7)	<.0001
Lymphoma	66 (7.1)	33 (4.2)	<20	2443 (3.7)	2558 (3.7)	<.0001
Bone marrow transplant	159 (17.0)	61 (7.7)	51 (18.1)	2081 (3.1)	2342 (3.4)	<.0001
Solid organ transplant	213 (22.8)	86 (10.8)	78 (27.7)	2946 (4.4)	3304 (4.8)	<.0001
Medications/medical devices^d^						
Dexamethasone	276 (29.5)	194 (24.4)	64 (22.6)	14 598 (21.3)	15 113 (22.1)	<.0001
Remdesivir	407 (43.6)	241 (30.3)	108 (38.2)	22 673 (34.1)	23 399 (34.2)	.01
Tocilizumab	98 (10.5)	59 (7.4)	20 (7.0)	4110 (6.2)	4283 (6.3)	<.0001
Methylprednisolone	85 (9.1)	72 (9.0)	20 (7.0)	2270 (3.4)	2435 (3.5)	<.0001
Central venous catheter	638 (68.3)	568 (71.6)	165 (58.5)	25 433 (38.2)	26 744 (39.1)	<.0001

Abbreviations: CAIFI, COVID-19-associated invasive fungal infection; COVID-19, coronavirus disease 2019.

^a^Percentages are column percentages.

^b^Other invasive fungal infections include cases associated with: histoplasmosis, mucormycosis, blastomycosis, *Cryptococcus*, and coccidioidomycosis.

^c^Chi-square test, Fisher exact test, or Wilcoxon rank-sum test was used as suitable. *P* value was calculated comparing individuals with no fungal infections with individuals with any CAIFI.

^d^We defined use of steroids as administration of each drug for 3 days or more. Per N3C policy, cells with <20 should not be reported with exact number.

The proportion of patients receiving dexamethasone (26.6% vs 21.9%; *P* < .0001), remdesivir (37.5% vs 34.1%; *P* = .01), and tocilizumab (8.9% vs 6.1%; *P* < .0001) was significantly higher among patients with CAIFI than those without (Table 1). Overall, 9.5% (n = 6544/68 383) received systemic antifungals; this proportion increased to 55.9% (n = 1082/1934) among patients with a CAIFI diagnosis.

### Incidence

The incidence of CAIFI exhibited multiple peaks throughout the pandemic, aligning with individual waves of COVID-19 variants. Notably, CAIFI incidence peaks consistently lagged 6–8 weeks following COVID-19 peaks (Supplementary Figure 8). To provide a visual representation of the temporal trends, Supplementary Figures 9–10 display the incidence of CAIFI associated with *Aspergillus* and invasive candidiasis over time, respectively. Incidence rates of other CAIFIs are shown in Supplementary Figures 11–15.

Intubated patients with influenza had a comparable incidence of IFIs as intubated patients with COVID-19. The 2 groups had no significant differences in the incidence of IFIs due to specific pathogens (Supplementary Table 5). Conversely, the incidence of IFI was higher among intubated patients with COVID-19 when compared with intubated patients with a diagnosis of RSV, though RSV incidence was very low (Supplementary Table 6).

In our cohort, 27 625 patients with COVID-19 and 34 602 without COVID-19 experienced prolonged intubation beyond 96 hours. IFI incidence was higher in COVID-19 patients (4.82% vs 4.06%; *P* = .01), including aspergillosis, compared with those with no COVID-19 (2.40% vs 1.39%; *P* = .01) (Supplementary Table 7). No significant difference in IFI incidence was observed between COVID-19 and influenza patients or specific IFIs in this population experiencing prolonged intubation (Supplementary Table 8).

Out of 68 383 patients, 6.5% (n = 4463/68 383) had a galactomannan (serum or BAL) test performed. The incidence of aspergillosis among individuals with a BAL test was 6.2% (n = 83/1328), whereas the incidence among those who had a serum galactomannan test was 2.8% (n = 88/3135). Moreover, among individuals who were never tested with galactomannan, the incidence of aspergillosis was 0.7% (n = 484/63 920). The incidence rates of CAIFI stratified by patients whose diagnosis was based on administrative coding, laboratory results only, and combined were statistically similar and are shown in Supplementary Table 9.

### Mortality

Patients with CAIFI had a higher 90-day crude all-cause mortality (50.9%; n = 985/1934; vs 36.8%; n = 24 487/66 449; *P* < .0001). Higher crude all-cause mortality at 30 days was also observed (36.5%; n = 707/1934; vs 32.7%; 21 737/66 449; *P* = .01). Pathogen-specific crude mortality proportions are reported in Supplementary Table 10.

**Figure 3.**
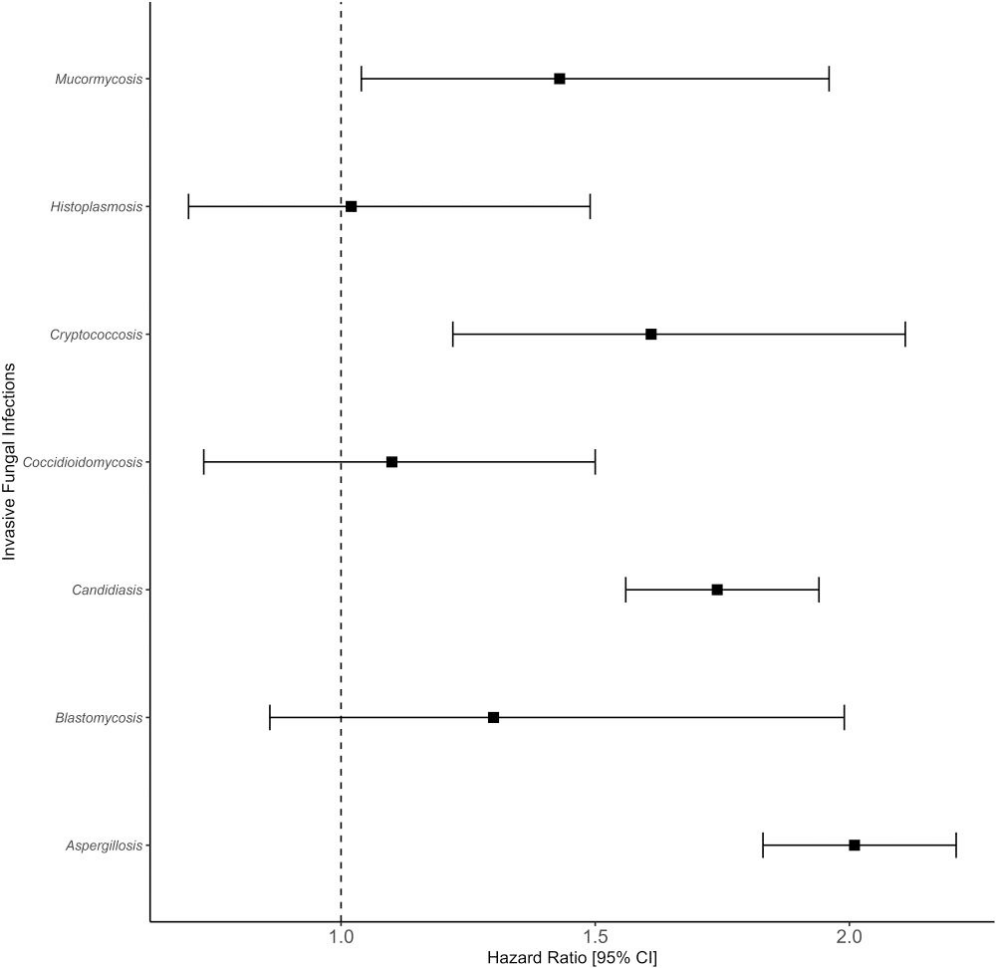
Association between CAIFIs and 90-day all-cause mortality among intubated patients within 76 centers in the United States (2020–2022). Abbreviations: CAIFIs, COVID-19-associated invasive fungal infections; COVID-19, coronavirus disease 2019.

After accounting for immortal time bias and confounders, infections associated with *Aspergillus* (hazard ratio [HR], 2.0; 95% CI, 1.8–2.2), *Candida* (HR, 1.7; 95% CI, 1.5–1.9), *Cryptococcus* (HR, 1.6; 95% CI, 1.2–2.1), and Mucorales (HR, 1.4; 95% CI, 1.0–2.0) were associated with increased 90-day all-cause mortality (Figure 3). *Aspergillus* (HR, 1.8; 95% CI, 1.6–2.0), *Candida* (HR, 1.2; 95% CI, 1.1–1.4), and Mucorales (HR, 1.4; 95% CI, 1.0–2.0) were also associated with 30-day all-cause mortality (Supplementary Figure 16). The survival analysis in Supplementary Table 11 shows a strong association between CAIFI due to *Aspergillus* and *Candida* with 90-day all-cause mortality among the 3 waves.

After sensitivity analysis, we observed an association between CAIFIs associated with *Aspergillus, Candida,* and *Cryptococcus* and 90-day all-cause mortality when accounting for diagnostic methods, including clinical diagnosis (Supplementary Table 12).

Following effect modification analysis, patients with CAIFI due to *Aspergillus* had a significant reduction in 30-day (HR from 2.2 [95% CI, 1.9–2.7] to 1.6 [95% CI, 1.4–1.8]) and 90-day (HR from 2.3 [95% CI, 1.9–2.7] to 1.9 [95% CI, 1.6–2.1]) all-cause mortality if they received systemic antifungals, when compared with patients without receipt of systemic antifungals. This reduction was also present within patients who had a CAIFI associated with *Candida* in both 30-day (HR from 2.0 [95% CI, 1.7–2.3] to 1.2 [95% CI, 1.1–1.4) and 90-day (HR from 2.1 [95% CI, 1.9–2.3] to 1.6 [95% CI, 1.5–1.8]) all-cause mortality (Supplementary Table 13).

## DISCUSSION

We reported a cumulative CAIFI incidence of 2.8% among intubated, critically ill patients from a database of >4.9 million patients from 76 medical centers across the US collected throughout the COVID-19 pandemic. We observed higher all-cause mortality at 90 days in patients with CAIFI due to *Aspergillus, Candida, Cryptococcus,* and Mucorales, even when adjusting for COVID-19 treatments, comorbidities, and other underlying characteristics.

The observed incidence was substantially lower than the 5%–33% reported in prior studies [32]. Lower CAIFI incidence was consistently observed across multiple subanalyses. For example, the incidence of pathogen-specific CAIFI was similarly lower than previous reports. The incidence of *Aspergillus*-associated CAIFI in our study was 1.3%. European studies reported up to 27% incidence [12], Brazilian data found 16% [7], and a large, multinational study noted a median 11% incidence [9]. Many of these studies, conducted primarily during early pandemic waves, may not reflect later variants like Omicron. Our analysis provides a broader temporal perspective, encompassing each COVID-19 wave. However, we did not detect a difference in CAIFI incidence between pandemic waves.

Due to the study's scope, we could not definitively evaluate factors contributing to lower CAIFI incidence. Scientific and medical advances (eg, vaccination) likely impacted patient outcomes and are difficult to adjust for in observational data. Still, we conducted extra analyses to contextualize the low CAIFI incidence observed in our study and its role in subsequent CAIFI mortality analyses.

The incidence of CAIFI consistently ranged from 1% to 4% across all our analyses. We delved into various aspects, including the pandemic waves, to explore associations with COVID-19 variants and advancements in medical interventions such as vaccine development. Additionally, we examined the impact of COVID-19 treatments, particularly considering the prevalence of immunomodulatory medications. Our analyses also considered the duration of mechanical ventilation, aiming to mitigate the influence of transient or less severe indications for intubation. Furthermore, we explored the diagnostic methodology to understand the relationship between laboratory testing and diagnostic/billing codes. In every facet of our investigation, the incidence of CAIFI remained below 4%. However, when specifically assessing the incidence of aspergillosis among patients who underwent galactomannan BAL testing, the incidence increased to 6%. We presented the incidence among both tested and nontested individuals in our study, following previous findings that suggested reporting the incidence of invasive aspergillosis in this manner. This approach is crucial as using the wrong denominator can lead to underestimating this infection. In the latter analysis, studies with higher testing rates almost doubled the median incidence compared with studies with low testing rates. Therefore, clinicians should proactively test patients with severe influenza or COVID-19, particularly in non-neutropenic populations. This proactive approach is essential for promptly diagnosing this infection, which might otherwise go undetected [33].

Each analysis was designed to address a specific clinical concern that could affect the interpretation of our data. Medical interventions for COVID-19 evolved rapidly during the early pandemic while awaiting data from clinical trials. Treatment regimens stabilized over time, and dexamethasone, remdesivir, and tocilizumab emerged as common therapeutic agents for much of the pandemic. While statistically significant differences were observed in the receipt of dexamethasone, remdesivir, or tocilizumab between patients with CAIFI and those without, their clinical relevance may be nuanced for the specific focus of this analysis. However, it is essential to recognize their role as confounders in our survival models, warranting their inclusion for accurate adjustment rather than emphasizing their direct clinical importance in this context.

Intubation status is a simple surrogate marker for critical illness but can be required for indications unrelated to respiratory infection (eg, airway protection). To minimize the impact of transient or unrelated indications, we analyzed only patients requiring intubation ≥96 hours. The small absolute difference in CAIFI incidence between the short- and long-duration intubation groups was clinically insignificant.

Finally, our use of administrative data may have affected the low CAIFI incidence observed in our study. Analyzing data derived from administrative coding can introduce misclassification bias. However, administrative databases are increasingly utilized because they provide extensive amounts of data, enabling research that would otherwise require lengthy periods and substantial resources. Our sensitivity analysis stratified between patients with a diagnosis based solely on administrative codes, based solely on laboratory results, and those with both. We did not detect a clinically significant difference in the incidence rates by the method of diagnosis. The consistency of CAIFI incidence across several exploratory analyses suggests internal consistency of our definitions and methodology.

IFIs, especially aspergillosis, have been associated with other respiratory viruses [34]. We compared IFI incidence in patients with COVID-19 vs patients with influenza. There were no clinically or statistically significant differences between CAIFI incidence in patients with COVID-19 vs those with influenza. Adjusting the analysis for intubation duration and specific fungal infections (aspergillosis and candidiasis) did not affect incidence rates. This sensitivity analysis should be interpreted as an exploratory analysis. However, it suggests that IFI susceptibility may be related to respiratory viral illnesses generally, rather than to COVID-19 specifically. Patients with RSV were also analyzed, though IFIs were rare, limiting further analysis due to a low fragility index.

Mortality in intubated COVID-19 patients with *Aspergillus* infections has been extensively studied, but the observed mortality rates are heterogenous. Permpalung et al. found no difference in intensive care unit (ICU) mortality among 39 patients [6]. A larger cohort identified an association but was limited by unspecified follow-up and single-center design [35]. An analysis in 20 centers showed higher 90-day ICU mortality in 109 aspergillosis patients and adjusted for immortal time bias [9]. While numerous investigations have delved into the mortality dynamics associated with COVID-19 and aspergillosis, this outcome has exhibited substantial heterogeneity. Moreover, investigations evaluating the efficacy of antifungal prophylaxis or antifungal therapeutic interventions for COVID-19-associated invasive aspergillosis have encountered challenges in delineating a definitive advantage for either approach [10, 12, 36]. It is crucial to acknowledge that our study did not explicitly delve into attributable mortality. While widely used, the terminology “all-cause mortality” might not comprehensively capture the intricate nuances inherent in this context. Nonetheless, our effect modification analysis showed that patients who received systemic antifungal therapy in our study showed a 28% reduction in 30-day all-cause mortality and a 17% reduction in 90-day all-cause mortality. This noteworthy finding raises the hypothesis of a plausible causal relationship between the presence of aspergillosis and mortality, suggesting a deeper interconnection extending beyond mere coexistence. These observations underscore the critical need for further research to uncover the intricate interplay of mortality in severe COVID-19 cases with associated aspergillosis.

Previous studies have not consistently observed benefit of antifungal therapy in patients with COVID-19-associated aspergillosis. Some indicate that antifungal treatment enhances survival [37], while others report varying degrees of benefit [10, 12]. Nonetheless, limitations in the latter studies hindered the accurate estimation of this effect: (1) limited sample size, (2) absence of confounder adjustment, and (3) utilization of less robust statistical methodologies. Addressing these limitations, our findings reveal a consistent and significant reduction in mortality with treatment, shedding light on the crucial role of timely interventions and contributing valuable insights to inform clinical practice and improve patient outcomes in COVID-19-associated invasive aspergillosis. A target trial emulation is a feasible approach that would improve our understanding of this critical question.

*Candida* spp. were the second most common CAIFIs in our extensive cohort (1% n = 793/68 383). The incidence in previous studies has varied greatly: Szabo et al. documented 40% candidemia among 20 critically ill Hungarian patients [38], whereas Gangneux et al. reported 6% in a French cohort [12]. Crude candidemia-associated mortality reporting at specific intervals is scarce [12]. In Turkey, candidemia drove 86% of in-hospital mortality within 28 COVID-19 patients [39]. Different 30-day mortality rates were observed in studies focusing on *Candida* auris, with 27% in an ICU [15] and 64% in India [16]. The geographic discrepancy in mortality largely mirrors candidemia-associated mortality in non-COVID-19 studies [40].

Our study identified 793 cases of invasive candidiasis, which, to our knowledge, represents the largest cohort of critically ill patients with COVID-19-associated candidiasis. Crude all-cause mortality was 29.0% at 30 days and 47.2% at 90 days. Further, we observed an association between invasive candidiasis and 30-day and 90-day mortality compared with patients without. Notably, antifungal treatment mirrored aspergillosis results: Mortality estimates declined, suggesting a causal link. This bolsters the significance of timely intervention against *Candida* bloodstream infections and the necessity of antifungal therapy.

COVID-19-associated mucormycosis first became prominent with the Delta wave in India [41]. In previous case series with mostly patients diagnosed in India, mucormycosis has been associated with higher crude mortality [17, 42–44]. To our knowledge, our analysis is one of the few to assess mortality for this infection by comparing it with a control group and adjusting for potential confounders. Predictably, we found an association between mucormycosis and 30-day and 90-day all-cause mortality among intubated patients with SARS-CoV-2 infection.

Our analysis of cryptococcal infections also revealed an association with elevated mortality. Our results align with a previous analysis that observed higher 90-day mortality in COVID-19-associated cryptococcosis [45].

As our analysis contained data since the pandemic started, we had the opportunity to evaluate possible differences in mortality within different COVID-19 waves. Mortality at 90 days remained similarly high among patients with CAIFI compared with those who did not present with a CAIFI, regardless of which COVID-19 variants were circulating during the different pandemic waves. Mortality also did not appear to be dependent on circulation of specific COVID-19 strains. Genomic analysis could elucidate this with more clarity.

Our study, conducted in an extensive multicenter database, is one of the largest to explore the link between multiple CAIFIs and mortality in intubated COVID-19 patients. We employed inverse probability weighting with doubly robust estimation to minimize confounding and provide a more precise estimate. We reduced immortal time bias by including time-dependent exposure in survival models.

A major limitation of this study is that our results are exclusively from patients with COVID-19 diagnosed with CAIFI at hospitals in the United States. Individuals interpreting our data should note this geographic limitation and consider their local fungal epidemiology. Further, due to data availability, our analysis does not provide an analysis stratified by each of the different sites that contributed to this database. Therefore, we were not able to determine the variability of both incidence and mortality among the N3C study sites. Our analysis includes data from 76 centers in the United States, which makes it subject to lack of homogeneity on how cases for each of the CAIFIs were diagnosed and their therapeutic approach. Variable clinical practices can impact both incidence and mortality.

We acknowledge the limitations of using administrative data, particularly in stratifying cohorts based on MSG/EORTC classifications of IFI. Those diagnostic criteria are optimized for use in patients with conventional risk factors for IFI (eg, immunocompromise). We refrained from employing the MSG/EORTC criteria (1) because most of the patients in our cohort lack conventional IFI risk factors (refer to Table 1) [46] and (2) because of the suboptimal performance of the MSG/EORTC criteria in other ICU cohorts [47]. Further, previous work has documented the difficulty applying the MSG/EORTC definitions consistently across various studies, especially when not all diagnostic testing modalities suggested by these consensus guidelines are available [48]. To address potential biases from administrative coding, we conducted a sensitivity analysis stratifying patients by diagnosis method—administrative code only or both code and positive laboratory result. Despite variations in the magnitude of the association within these groups, a robust link between CAIFI and all-cause mortality at both 30-day and 90-day intervals persisted.

In conclusion, our study showed a relatively low CAIFI incidence in the United States but a solid association with increased mortality among intubated patients with COVID-19. Clinicians should maintain a high degree of suspicion for CAIFI and intervene with appropriate antifungals when appropriate.

## Supplementary Material

ofae108_Supplementary_Data

## Supplementary Data

Supplementary materials are available at *Open Forum Infectious Diseases* online. Consisting of data provided by the authors to benefit the reader, the posted materials are not copyedited and are the sole responsibility of the authors, so questions or comments should be addressed to the corresponding author.
