# Supplementary material for: Incidence and Mortality of COVID-19-Associated Invasive Fungal Infections Among Critically Ill Intubated Patients: A Multicenter Retrospective Cohort Analysis
Source: Open Forum Infect Dis. 2024 Apr 2;11(4):ofae108. doi: 10.1093/ofid/ofae108 (PMC10986750; doi:10.1093/ofid/ofae108)
Supplement: ofae108_Supplementary_Data [file ofae108_supplementary_data.docx]

Appendix Methods. Statistical Analysis

The incidence of IFIs was examined in both COVID-19-positive and COVID-19-negative patients, utilizing proportions as a means of representation. A sensitivity analysis was conducted to explore the incidence of IFIs, specifically among patients intubated for a duration exceeding 96 hours. Patients intubated for a shorter time period were at much lower risk for developing complications.

We analyzed each CAIFI individually. The control group was patients without any CAIFI diagnosis. We used stabilized weights to adjust for confounding via inverse probability weighting (1, 2). Elixhauser comorbidities, historical IFI risk factors, and clinically plausible risk factors (chosen *a priori*) were used for adjustment [Appendix Tables 1-2]. Continuous variables were assessed using restricted cubic splines. We built a separate inverse probability weighting analysis for each of the different CAIFI. We fitted a logistic regression model in which each CAIFI was the exposure variable and each of the different covariates as predictor variables. Following this, we calculated predicted probabilities to calculate stabilized weights. Each of these weights were later used in each of the separate Cox survival model. After conducting inverse probability weighting for each CAIFI, the balancing of the baseline characteristics was assessed using standardized mean differences (SMD) and considered adequately balanced if the SMD was below 0.1 (3) [Appendix Figures 1-7].

Cox proportional hazard models assessed 30-day and 90-day all-cause mortality for each infection. Stratified analysis was conducted for pandemic waves (Wuhan-HU-1, Delta, and Omicron) (4). Time-dependent exposures were used to reduce immortal time bias (5). CAIFI was the sole explanatory variable if baseline covariates were balanced. Doubly robust estimations were applied for unbalanced covariates (SMD > 0.1) (6).

Sensitivity analysis compared ICD/SNOMED-CT codes and lab testing for 90-day all-cause mortality, while effect modification by treatment status was analyzed. Cox models assessed each CAIFI as an exposure and 90-day mortality as outcomes. Treatment for IFIs adhered to relevant guidelines (7-11).

Data management and preparation for analysis were performed using Spark SQL (Apache-spark.org; version 3.0.2), while the descriptive analysis and survival analysis were performed using SparkR (r-project.org; version 4.2.1) within the N3C database.

Appendix Table 1. Medications associated to COVID-19 treatment

| Medication | OMOP Concepts | Standard Vocabulary Codes |
| --- | --- | --- |
| Remdesivir | 3658363, 32763, 37499275, 37002796, 3574635, 1146730, 1145690, 3666998, 37003601, 37499271, 37499274 | *RxNorm:* 2284960, 2395502, 2367758, 2367759, 2395504, 2284718, 2284959, OMOP4873974**    *SNOMED: 38377611000001105, 38376211000001106, 870592005* |
| Tocilizumab | 46275124, 45341907, 44983658, 40171290, 46275121, 44847503, 44507562, 46275116, 45290963, 40958504, 46275122, 46275123, 36496757, 46275118, 45136890, 45017822, 40171288, 45136891, 44507566 | *RxNorm: 1657982, 895760, 1657979, 1441530, 1657974, 1657980, 1657981, 1657976, 612865, 1441527, OMOP2156466***    *NDC:* 50242013504, 50242013501, 50242013601, 50242013801, 50242013886, 50242013701, 50242013604, 50242013704 |
| Dexamethasone | 45361633, 36180492, 35526658, 45040458, 42839524, 37298569, 35524857, 45156666, 45865388, 45867460, 42508528, 35837987, 45125418, 973177, 36187302, 44426479, 36900510, 1997771, 44865910, 35838075, 36180493, 45364346, 36117578, 44984884, 45852768, 45835739, 42829922, 35300196, 45213483, 42829923, 45193639, 1626224, 45903198, 45852714, 36187304, 45240827, 35204412, 44479309, 42839542, 45869182, 45037500, 771884, 35301159, 44917195, 44957615, 44429218, 44444866, 1629793, 45070323, 44464772, 45364496, 45852712, 44904454, 35999001, 42829924, 45869581, 45262516, 45074840, 42631340, 1765925, 45279767, 36194483, 36180489, 750522, 45867087, 42508526, 45373793, 45203117, 44420389, 1565739, 43559087, 44479310, 37603657, 36381277, 42830571, 1630229, 42841194, 45088491, 44430026, 45863321, 45053181, 45178997, 45025887, 44781365, 45875626, 44444538, 42841191, 44428859, 35403493, 1228259, 1779695, 45075316, 36117580, 45075649, 44429538, 45380632, 45331458, 42844619, 45254562, 35504881, 44955220, 45332870, 45241865, 45223738, 45852716, 45864159, 35519343, 36496135, 42661392, 45867907, 1779544, 1997768, 759059, 36116532, 45228114, 1528606, 1161647, 45023596, 45315934, 45057459, 1997765, 45025886, 45068073, 36398440, 36180494, 1583228, 36185200, 44884292, 45329085, 44922136, 1743536, 44972434, 36800463, 35899015, 44853135, 36480712, 45339778, 45100781, 44871111, 45207904, 726523, 36489156, 44906982, 1997770, 36117575, 35999525, 45867085, 44955453, 44427380, 36479320, 35109914, 1753241, 1190048, 36501220, 36187299, 45054653, 36109441, 42830569, 45125281, 1997766, 35403045, 44781114, 36187306, 36177448, 45111085, 35519341, 45068949, 737739, 44841173, 45211126, 44853325, 35519344, 44478460, 1629791, 822445, 45057304, 36187301, 44487789, 44947735, 737543, 46331657, 45862945, 35601657, 35520208, 35519967, 44423370, 36187300, 45275113, 35402960, 750519, 45053204, 1228258, 44417608, 1997773, 44923994, 737740, 45905101, 46368032, 35519968, 45109648, 46249118, 44436107, 36134575, 45074639, 36177450, 36391536, 46255536, 36187303, 45902858, 1779457, 42828794, 45230747, 42654843, 42830576, 44923880, 42884391, 1225182, 45041201, 45335815, 1788888, 45164466, 46331994, 44907103, 1997774, 45262303, 45228105, 797033, 35838845, 44949692, 36134574, 36191533, 42844618, 771885, 46331594, 44439630, 36117576, 42644298, 1223595, 44436601, 44884696, 45280392, 42841190, 36478884, 44438277, 1228264, 36601879, 36178902, 44436877, 45083828, 46329976, 1185596, 36398453, 45094114, 42830568, 42844616, 45296701, 36180490, 36180487, 45108586, 46367887, 45068948, 35402660, 45262518, 44922440, 45230746, 42830572, 35402799, 43553168, 44422722, 45091734, 44969313, 36194484, 45331118, 42830570, 1228261, 593652, 750520, 45196086, 1228263, 45109404, 45347471, 45060167, 796645, 45281913, 1228262, 45904822, 42631339, 36117577, 45040416, 35519965, 44781366, 45210748, 42830575, 45181328, 45225228, 45318262, 1105285, 35519339, 45150819, 44438376, 35524856, 1143984, 1630228, 42829920, 45867908, 45159658, 36116119, 35839774, 44466675, 44420312, 37300408, 1142041, 42508527, 42519796, 1630231, 44918728, 36109442, 42829921, 44972223, 1190049, 44426478, 1171652, 45042925, 1171653, 42830573, 44781367, 45254678, 42635636, 44906971, 43559088, 44900138, 35519342, 44966391, 599972, 1997767, 45006914, 45327604, 44907126, 1161920, 44882996, 45280078, 36134573, 35108350, 819853, 36177449, 726392, 44497460, 1228260, 35402887, 44906999, 45863797, 36180491, 36180488, 1626349, 42844617, 44951055, 44865876, 44410693, 36194485, 35519340, 36900242, 44415101, 1142042, 35519345, 42830574, 44434374, 44981511, 35519892, 1358082, 42508048, 45281787, 42841193, 44989848, 1528907, 45903528, 44957618, 35403040, 1162491, 45276584, 44479308, 42839562, 42830567, 45246176, 750521, 44448935, 44957628, 1233726, 36479029, 1779662, 44462099, 36117574, 46368602, 45196093, 36479419, 45365178, 46364981, 45037571, 45262517, 44439631, 1997769, 36104001, 1997772, 44464168, 35403512, 44986416, 46331298, 45229149, 45867086, 45852711, 44424715, 45040658, 44951054, 45279575, 35204221, 42841192, 36184773, 1228265, 45852715, 36117579, 42635592, 36380334, 36187305, 44983548, 46331565, 45040473, 44412316, 35601520, 45074465, 43553167, 45091529, 737549, 44435257, 44410975, 45125614, 45298951, 45111207, 1779504, 45314500, 42519795, 1779564, 45144963, 1753240, 36177451, 35519966, 42828826, 40231785, 1518259, 19030003, 19029973, 19030008, 19024531, 1518261, 19076145, 19076135, 19006962, 19030005, 1719012, 19018906, 19095136, 1719046, 1518254, 40173366, 40241504, 19076136, 1518292, 19095151, 1518258, 1518293, 1719022, 35202020, 40028260, 19108508, 35203362, 1208799, 44386526, 44396133, 37295968, 42886633, 736837, 42816578, 45796728, 36901035, 45781604, 1743869, 35529809, 36169394, 35504067, 736824, 42660091, 42881464, 36142527, 42886649, 36870267, 42817103, 1767657, 35517148, 35837144, 45801615, 36869772, 44385320, 36114566, 42815408, 36141752, 821395, 42886682, 1357236, 35998445, 726813, 35116329, 1206038, 42886023, 1527597, 36163518, 42506549, 44403102, 42816599, 1629657, 1217517, 36869773, 36469346, 35501615, 1628479, 44384680, 796471, 42631892, 36801592, 42810388, 44380168, 35600855 | *RxNorm:* 1049400, 197579, 205712, 205702, 205717, 201072, 197581, 309696, 309684, 105392, 205714, 1812079, 197580, 343033, 1812194, 3264, 901649, 1116927, 309686, 197582, 343040, 197577, 197583, 1812095, 2099700, 371725, 436510    *NDC:* 55154491400, 70069002325, 76045010910, 63629374202, 718727021, 50090231303, 63629780602, 55289058228, 63629374200, 63629374209, 52584023805, 51662129702, 63629374201, 51662129701, 70121145005, 33261062515, 705182936, 71335210803, 52959150401, 76045021000, 70069002401, 63323051610, 48102005201, 52959039221, 674570484, 545694728, 71335007703, 68071274401, 00069017701, 71335007704, 63629374204, 81565020201, 50090009100, 674570421, 70121145105, 52959054716, 71872715701, 551500238, 718727092, 67457048400, 55154490100, 72572012001, 66993073051, 52959039212, 00054317644, 33261062521, 53217031001, 004049843, 52959039230, 50090062701, 63629412703, 674570419, 63629412704, 764200185, 71335007705, 63629374208, 63874079512, 63874079501, 66993073002, 71872717101, 64980050924, 701211450, 70069002201, 63323016503, 67457041805, 63629785003, 33261062510, 33261062502, 70518062100, 50090008800, 69306011160, 551500239, 55150030510, 63629412906, 71335017704, 00404984105, 71872702101, 551542733, 76045010610, 51655001203, 52959039228, 00054818025, 00054418425, 70529004501, 67457041800, 55150023701, 58463001501, 67296109001, 63323050616, 68071232105, 47781091413, 66267006720, 70518311900, 67457042300, 55150023805, 60432046608, 67457042000, 71872712801, 33261055860, 500901922, 63629374207, 00054817525, 54879000308, 52959054711, 674570423, 63629412905, 52959054707, 70518084300, 68788744101, 68151502604, 68083047301, 71335210806, 71872716401, 63323050641, 63629412702, 516621432, 70069002501, 63874047104, 00054818325, 63629374203, 71335210809, 00054417925, 43063026607, 00121090705, 70069002425, 51662134301, 53217037410, 551542732, 58468021802, 66267006630, 68788714206, 63874079530, 81565020202, 72189025410, 63629412904, 68071412702, 33261062530, 33261062560, 67457042200, 55154511805, 705183379, 53225366001, 00054418325, 71335210804, 48102005120, 76420018501, 67457041901, 63874079510, 63187038330, 68047070201, 69306011430, 63629785002, 00121090740, 70518337901, 70121139901, 55289058204, 51662139101, 71335017702, 63323050601, 71335210808, 63187056160, 49999005912, 70121145205, 48102004501, 00054817925, 52959054701, 49884008601, 71872714701, 00641036721, 63874079515, 63874079520, 52959054718, 525840422, 004049841, 70518301901, 63323016530, 70121145001, 680711866, 33261062590, 718727147, 61919026921, 67457048430, 76420007701, 70518153400, 68788726702, 00641614501, 70121139905, 52959054750, 63323016516, 68788714201, 52959054704, 68071232101, 70518053200, 71335210801, 000690178, 71872715301, 51655001287, 551547075, 68788726703, 67457042100, 10544021206, 33261055821, 60219204401, 63629412901, 48102004701, 69306011406, 10544021110, 70121145101, 50090008900, 68083047401, 70518053201, 000694547, 45865098906, 71335017709, 00054418625, 500902313, 48102004711, 66267006710, 00641036725, 50090354100, 005174905, 52584042010, 00069017702, 71335210800, 63629412902, 63629374205, 52584023930, 705183019, 49884008501, 60219204301, 500903300, 71872709201, 72572012201, 55154707505, 63187038305, 48102005140, 61919082715, 760450210, 49884037301, 55887037710, 67457042254, 58463001401, 68071412706, 63187056110, 51662129703, 50090456700, 63323016502, 52584042200, 33261055890, 55700026321, 63629412708, 00121181410, 000690179, 71335017701, 71872709101, 63874079540, 70069002225, 70069002101, 63629412706, 525840421, 49884008401, 63187056130, 63874079528, 67457042312, 00069454101, 71335017705, 63187056100, 55150030425, 55150023930, 63874079550, 55289058221, 701211451, 66267006721, 71335017703, 67296141302, 42195015110, 68788714203, 00054317757, 70518299800, 66336047940, 63629412705, 00069454301, 525840239, 00069454701, 67296141304, 50090009004, 66993073080, 48102004901, 63629269601, 68788726700, 70529004502, 63323016505, 71335017708, 005174930, 55289058210, 005174901, 61919082721, 52959039206, 13985004329, 00641614625, 63629780601, 55700096112, 00404984210, 71335007701, 67457048300, 63874079521, 705183119, 70518301900, 50090192200, 53217023190, 70518305000, 68083047425, 63187056115, 70069002510, 00404984330, 55289090310, 51662137001, 71335007702, 63629412903, 00121181440, 33261062506, 72572012225, 00054817425, 72572012025, 71335017706, 70529004505, 35356035930, 70518311901, 00054317763, 69306011230, 52959054710, 52959054705, 49884008701, 51662154101, 71335210807, 66267006708, 55289058206, 000694545, 551548338, 52959054712, 66267006612, 55289058215, 55150030401, 71872723901, 48102004601, 70518337900, 760450106, 67296141301, 63187056190, 00054818125, 67457048310, 70069002301, 70069002125, 70518293601, 71872709001, 52959054730, 52959054720, 53217023130, 701211452, 52959039215, 70518293600, 00641614525, 68083047325, 52959054728, 71335017707, 63629412707, 33261055830, 68071458001, 71872720501, 525840238, 00054418125, 58463001701, 66267006704, 51662143201, 50090008804, 00054418025, 63187056124, 55154833805, 55289090320, 551500237, 718727128, 71335017700, 67457042130, 68788714205, 53217023121, 00054817625, 50090062703, 68071412701, 47781091401, 50090062702, 48102005101, 617860372, 00054418231, 68071412705, 66267006712, 52584042100, 55154936405, 63874079524, 63187038390, 71335210805, 55150030501, 71335210802, 50090231302, 63323016526, 55289090312, 55700026355, 67296032601, 67457041900, 674570418, 00641614601, 63874047105, 52959054721, 63874079514, 718727157, 58463001601, 50090330000, 76045021010, 674570422, 48102005001, 48102005111, 48102004720, 70121145201, 49999005906, 61919026955, 63629412701, 63187038324, 764200077, 63323016501, 51407036101, 63629374206, 718727153, 53217023160, 63187038360, 63874047110, 00054418225, 000694543, 47781091451, 67457042010, 70518153401, 47781091601, 000690192, 63629785001, 48102004801, 68788726701, 70518087200    *SPL:* 86090e3c-9fd5-2fcf-e053-2a91aa0a6897, 916ef348-1e5b-40b4-9d12-508fc2ce567c, 581655f5-233d-31de-e053-2a91aa0ae0e2, 995257fc-7f80-464c-bf82-2c984d474398, 8affea41-497c-4bf5-999c-7541ba47e349, 9ce56830-67b1-bc93-e053-2a95a90aecf7, 81a28bdb-b187-8047-e053-2a91aa0a4e0a, 69346236-8fd5-1d49-e053-2a91aa0a5228, b41e2831-bc64-47bc-bb33-ddc8e2e77339, d4232eec-5c69-e488-e053-2a95a90a1743, 2217dd41-a75e-42b4-aafb-f9d7d2e44f42, e61de106-cc66-424e-bf0b-ed2443667dd3, 1e903680-ab19-481b-8d68-2062fb03ee6b, 74fa9651-c3d3-49a7-ac26-4db9e76c49a8, aafdb5ea-9a19-4656-b727-2ba43505ba92, 81a0503c-39ff-1c67-e053-2a91aa0a5b8f, 694bf57c-df6b-4465-a1b0-6285fc4f336f, 4769520b-ab4b-4fbe-aec7-daa0a497c141, fee99bdf-0ba4-436e-838a-18dbb67c806e, 9ce56cdc-3510-1b2d-e053-2995a90ad6bc, 0ce47e9e-7c98-40d3-a9b1-0f262cd4aaab, 7359afc0-4d5a-48e5-9fb4-2f0fb07da84f, b8eabb99-8ec5-40d0-aa86-07d6e8d6090c, d72bdde5-8a64-4b9f-a8c7-fd132374e5a1, bad5a27d-2fb1-c318-e053-2995a90ad604, e4b6c252-64be-4e4a-88ba-3491d8fd7b3d, e5335fde-7f9a-6f54-e053-2a95a90ae881, 56426d41-81ca-4b49-9a9d-07b74ec706a1, c4bdbedf-24d4-e9de-e053-2995a90ad835, 667323ea-5a5f-3465-e053-2a91aa0a1667, c3a27c07-8f25-489a-af63-2f80f29f050e, 6b0ee0f2-08e5-4090-a7fd-44ff5b9739ca, 9ce59e9a-1233-4ebf-e053-2995a90aa786, 9f34beae-ebbe-2ba3-e053-2995a90affaf, b4ea9bea-3f7b-4dc9-e053-2a95a90ae557, d9cb24e4-0925-b8eb-e053-2a95a90a67ab, 02bf5ccd-597e-4731-ae4e-3eeb79f11d78, 1f33deb0-c256-4ddb-b031-df62fdaa6ffb, 0d7e3c77-2e0b-46ec-86da-bb03e37e4ae7, 9a426a16-19f9-567e-e053-2a95a90a0a5b, 5d284479-1d64-4cf7-94d4-589f412493d3, 1dde8e4c-c4ee-47a0-9e77-66b79ab57fb5, d42966db-1587-4973-8c65-4acbc091ce11, 69485218-9331-952d-e053-2a91aa0ad4e8, df7c8dd5-3828-4bab-b8a2-a92b6fe106c1, d5b59e4a-4050-4632-85a5-3e6adb011972, e5333c2e-03d1-23bb-e053-2a95a90ade22, 22bc1cad-e307-4369-a461-e5370df5b102, 4c1a6269-b693-454e-8554-e8fad49e2444, 9662adc1-3b57-40ac-ac77-ec2239b13818, 553accf9-f63d-3b80-e054-00144ff88e88, b84da20a-9dd5-4fea-be4d-3384aa4e5abd, dd1815f7-a5b7-33c8-e053-2995a90aa8d2, 9df908d4-8a77-46c9-a1db-67c8078899c8, 4dae51f7-b0d1-4a7d-afd8-9f5c628c3711, 4a9a8f85-883a-4365-979e-eb6a9ca807fd, 9e47c16d-897e-118f-e053-2995a90a24f1 |
| Methylprednisolone | 1743285, 44414177, 42644655, 36398476, 44437294, 36415108, 2519227, 729549, 42635715, 36179878, 43547690, 44929049, 45198086, 45298652, 44977517, 36182986, 44903306, 1994711, 36178379, 44991267, 37319936, 45331714, 45107359, 45863686, 36181035, 45352627, 36179449, 44442476, 44445241, 36999406, 1171803, 36179867, 36415107, 44409177, 45359598, 35500208, 45351992, 36181024, 1743283, 44421396, 36181242, 36179875, 36398459, 45155107, 45122497, 1583376, 1541138, 45042599, 45093705, 36182985, 45074628, 36181034, 36179877, 44423796, 36196627, 36104198, 36999405, 44431064, 35506089, 44976652, 1623159, 45122717, 44425156, 44423790, 44940604, 1541418, 36181023, 45213063, 36196628, 44924972, 1565775, 42631210, 1743282, 748163, 45034121, 36197982, 45331206, 45250174, 1994710, 45869824, 1630437, 1753409, 44934052, 43547691, 1623148, 45300969, 35500210, 45363328, 42644658, 44874634, 750786, 45341128, 44863824, 45127828, 44439085, 45315640, 45195272, 45244985, 44986475, 36179866, 44943083, 1743284, 45210755, 36179874, 44850227, 45867713, 36181032, 45175417, 45147098, 1623280, 36415106, 44430624, 1225919, 1623321, 44443163, 36182987, 44436415, 1541419, 45247262, 44906649, 42828780, 45059740, 45054514, 45213067, 45247260, 35500207, 36179412, 44949070, 37300308, 45264485, 829828, 45093719, 44923579, 1753410, 36009005, 45088662, 36181025, 44939720, 45366591, 36415110, 35500206, 37317631, 35500209, 44966467, 44435712, 36773705, 36181033, 36179876, 45159311, 36415111, 45005038, 45155119, 44840506, 44892998, 1541417, 35531217, 36184254, 45349655, 37300309, 1541139, 45327466, 45216539, 824374, 36501221, 36197981, 36602038, 1623223, 42827759, 1225918, 36181031, 1997601, 1630436, 42901998, 42901997, 19034807, 35606533, 1506479, 35606536, 1506426, 19034647, 1506430, 19007003, 35606542, 19034806, 19099970, 35606544, 35606538, 19034805, 1506270, 19034804, 35606540, 19080181, 19067555 | *RxNorm:* 1357888, 1357886, 207193, 1743704, 328161, 1743707, 259966, 207138, 311659, 105403, 1743726, 207192, 360536, 1743729, 1743720, 207191, 6902, 207190, 1743722, 314099, 260330    *NDC:* 43598012925, 70121100105, 68180068808, 71329030201, 63739013310, 50436000101, 00009085001, 70518202300, 70518312801, 68382091877, 51662126302, 10130021801, 00381072950, 00009075801, 00641250841, 00009004704, 59762444003, 43598012901, 57582010101, 00009069801, 70518312800, 68084014901, 59762005001, 54771354701, 70771135007, 00603459321, 68071402801, 25021081030, 25021080810, 51662126403, 72647033101, 68382091605, 65162087110, 50090027100, 51309040450, 71329030501, 00409568502, 70771134803, 43598012725, 55150026203, 71205005421, 68382091805, 71329030101, 52584004725, 55154394105, 42806040001, 82094040450, 00009003933, 00009003932, 00009003906, 63629391001, 70771135005, 68382091830, 70385201601, 00009004703, 70625023701, 51662126303, 50090027101, 57582010102, 00409568401, 82094040101, 55390021901, 70121100001, 70121100101, 00009001820, 82094040150, 70771134801, 00009004722, 00009003905, 00223816304, 50742018901, 71872708501, 43598012811, 70518202302, 45865041760, 70518142400, 66267096121, 00591079001, 43598012745, 61699004702, 00404995801, 00143985101, 52584003930, 51662126402, 82094040501, 00409321705, 71329030225, 59746000314, 68180068601, 00409321805, 71872706101, 45865041790, 45865041751, 00052078216, 55150026420, 00009079601, 68788459302, 63323026530, 55390021801, 68382091601, 00469129060, 43598013074, 63323025503, 68382091801, 55289064930, 55154394405, 70771135002, 59762332702, 00469188008, 82094040401, 65162087140, 70121100005, 45865010421, 82094040301, 55150026303, 00009069802, 55150026550, 82094040550, 00009005602, 00009003928, 70518033400, 00009004727, 55154394005, 00009007301, 00009004726, 71329030125, 68071179202, 45865041730, 69306040021, 00009003930, 63629221801, 00009091105, 00009000302, 00143985001, 70518204100, 55289064998, 70771134805, 68084014911, 00009004725, 65162098229, 71329030301, 68071522101, 71329030401, 49999015330, 25021080705, 70518294200, 70771135004, 68382091818, 63323025803, 65162098205, 59746000106, 52584072950, 00469104060, 00781502210, 82094040350, 00904691461, 45865041721, 00009091101, 68071237001, 82094040250, 55154393905, 00781502201, 63629243301, 70518202303, 53002312001, 70518202301, 82094040201, 68071426201, 71872723201, 70771135001, 68071274501, 0040499570 |

*All medications were filtered to oral, intramuscular or intravenous administration routes. Remdesivir and tocilizumab were filtered to any administration of one or more consecutive day following COVID-19 diagnosis. Dexamethasone and Methylprednisolone were filtered to 3 or more consecutive days of administration following COVID-19 diagnosis. Standardized codes are RxNorm (<https://www.nlm.nih.gov/research/umls/rxnorm/index.html>), FDA Standard Product Labeling (SPL) codes (<https://www.fda.gov/industry/fda-data-standards-advisory-board/structured-product-labeling-resources>), National Drug Codes (<https://www.accessdata.fda.gov/scripts/cder/ndc/index.cfm>) , or SNOMED. Remdesivir is the least standardized, and appears in the source data in several forms.

** This is an RxNorm Extension Code.

Appendix Table 2. Pre-existing Medical Conditions Diagnosis Codes

| Condition | SNOMED Code | ICD-9 Code | ICD-10 code |
| --- | --- | --- | --- |
| Cardiac Arrhythmia | 17366009, 440059007, 251164006, 17869006, 287057009, 71908006, 195083004, 282825002,16797001,441509002,67198005,28189009, 195042002, 5370000, 6456007,  426749004, 63593006, 195080001, 195105007, 15964901000119100, 27885002, 44808001, 251175005, 66657009, 251167004, 233917008, 29717002, 361136003, 25569003, 720448006, 44103008, 12026006, 60423000, 111288001, 36083008, 698247007, 49436004, 48867003 | 427.x, 426.x, 427.3x, 427.4x, 427.6x,426.1x, 427.6x, 427.4x, 427.8x, V53.x, V45.x | I48.x, I49.x, R00.8,  I44.x, I45.x,  I49.5, I48, Z95.0,  I47.x, R00.1, |
| Drug Abuse | 191865004, 191900006, 191850000, 77355000, 363101005, 191849000, 191816009, 14784000, 191851001, 153501000119105, 11061003, 191839003, 268640002, 1471000119103, 191492000, 191833002, 191821007, 191483003, 191838006, 87858002, 418475009, 191493005, 429672007, 425533007, 275471001, 191486006, 38247002, 31956009, 70545002, 75544000, 64386003 | 304.x, 305.x, 292.x  304, 292 | F19, F18, F15, Z72.2, F11, F16, F12, F13, F14 |
| Fluid and Electrolytes Disorders | 190878005, 267448003, 76314005, 267445000  55004003, 35633007, 267446004, 267447008  71741003, 76220009, 72442006,237840007  34095006, 21639008, 75934005, 816082000  14140009, 43339004, 21420006, 51387008 | 276.x | E87.X, E22.X, E87,  E86 |
| Renal Failure | 365399009, 105502003, 161693006, 385971003,194781004, 66052004, 16726004, 700378005, 129161000119100, 285841000119104, 49220004, 194780003, 275408006, 700379002, 431857002, 698591006, 8501000119104, 104931000119100, 66610008, 431855005, 433144002, 431856006, 433146000, 737295003, 709044004, 46177005 | V56.3X, V56.3X, V56.X, V45.X, 404.9X, 404.0X, 404.1X, 403.9X, 403.1X, 588, 585.X, V42.X, 585, 586 | Z49.X, N18.X |
| Alcohol Abuse | 35637008, 161466001, 212813006, 66590003, 61144001, 57346004, 420054005,  29212009, 83521008, 212809004, 73097000, 191802004, 281004, 41083005  82782008, 9953008, 191480000, 719848005, 67426006, 7916009, 41309000, 50325005, 7052005, 2043009, 418279001, 418186002 | V11.3, 291.X, 571.X, 303.9, 980.X, 303, 265.2, 357.5, 425.5, 571, 535.3, 305 | Z71.X, Z50.2, Z72.X, K29.2, K70.X, F10, T51.X, I42.6, E52, K70.3, T51, G62.1, |
| Leukemia | 161436008 ,37810007,  17788007, 63364005, 77430005, 24072005, 103688009, 128931003, 22331004, 22197008, 32280000, 87163000, 51092000, 315739008, 110002002, 92812005, 277602003, 91861009,  154587007, 188725004, 188726003, 445448008,  188754005, 444911000, 188744006, 404136008,  93143009, 93451002, 277619001, 110004001,  413442004, 277473004, 277571004, 277589003,  92814006, 127225006, 277567002, 118613001,  188718006, 269632001, 95210003, 91857003,  445227008, 110007008, 92818009, 91855006, 188732008 | 204.1, 205, 208, 204, 202.4, 205.1, 207,  202, 207.2, 206 | C91, C91.X, C93.X,  C95.X, C92.X, C94.X, C94, C95, C92, C90.X, C93 |
| Chronic Pulmonary Disease | 45145000, 196053000, 13645005, 37471005,  40122008, 424643009, 74417001, 195951007,  195953005, 87433001, 57686001, 74015002,  196017002, 52333004, 17996008, 25897000,  69339004, 274096000, 48347002, 50043002,  4981000, 286964001, 90623003, 733858005,  389145006, 32398004, 73144008, 67242002,  78723001, 426853005, 68328006, 805002,  63480004, 22607003, 196001008, 196049002,  266361008, 13151001, 846635004, 12295008,  195977004, 87837008, 85761009, 29422001,  86638007, 62371005, 61937009, 17385007,  14700006, 8247009, 18690003, 51277007, 13394002, 195967001 | 492.8, 506.4, 502, 504, 508.X, 490,  503, 496, 416.X, 501, 505, 495, 500, 493, 493.X, 491, 494, 492 | J43.X, J67, J44.X, J66.X, J67.X, J68.X, J44.X, J65, J66.X, J40, J41, J61, J63.X, J64, I27.X, J66, J45.X, J63, J70.X, J43, J41.X, J44, J62.X, J45.X, J62, J42, J60, J47, J45, J46 |
| Depression | 765176007, 430852001, 76441001, 18818009,  36474008, 191613003, 782501005, 231485007,  78667006, 191627008, 268658008, 75084000,  55668003, 11806006, 73867007, 84984002,  192063005, 15639000, 68019004, 57194009,  231504006, 79298009, 47372000, 9674006,  36923009, 40379007, 47505003, 192051000,  66344007, 832007, 87512008, 191616006, 17226007 | 309.2X, 309.8X, 309.X, 296.X, 311, 300.X, 309.X, 309 | F32.X, F31.X, F33.X, F33, F32, F41.X, F43.X, F20.X, F34.X, F32 |
| Peripheral Vascular Disease | 51274000, 34881000119105, 52403007, 13954005, 399957001, 155431008, 266320008, 72092001, 400047006, 63491006 | 440.21, 440.2, 440.23, 443.82, 443.8, 443.9 | I79.2, I73.1, I73.8, I70, I70.2, I73.9 |
| Valvular Disease | 449843004, 416940007, 29928006, 8722008, 18687009, 194741006, 60234000, 67696008, 56819008, 195012000, 787001, 194995005, 195005009, 18546004, 195004008, 16440002, 409712001, 195000004, 703178004, 195002007, 48872007, 48724000, 708121009, 195003002,194997002, 274097009, 703320000,  703319006, 49915006, 194987006, 28656008,  194989009, 23685000, 78031003, 88318005,  49699002, 194727002, 194732001, 194726006,  82458004, 194983005, 195013005, 17759006,  83898004, 12023003, 739026008, 16063004, 56786000, 315615007, 76267008, 111287006,  91434003, 368009, 67391006, 31085000, 72011007, 20721001, 301105002, 86466006, 89736004, 79619009, 11851006, 60573004, 123596001 | V43.3, 93.2, 395.9, 395.1, 394.2, 395.2, 746.4, 397.1, 394.1, 397.9, 424.2, 746.5, 394.9, 424, 397, 746.3, V42.2, 424.1, 396, 746.6, 395, 394, 424.3, 395, 424, 394 | Z95.3, I08.3, I07.2, I06.9, Q23.0, I37.8,  Z95.2, I39, I08, Q23.2, I06.2, I07.0, I37.0, I08.8, I37.2, I39.3, I07.8, I09.1,  I35.2, I37, I37.1, I34.9, I34.2, I35.9, I39.4, I05.1, I05, I07.9, I05.0, I06.0, I37.9, I39.0, I34, I05.2, I08.1, I05.9, Z95.4, I08.0, I39.8, I06.1, Q23.1, I34.8, I39.1, I09.8, I39.2, I07, I05.8, I06.8, I34.1, I08.9, I35.0, I34.0, I35.8, I38, I08.2, I07.1, I06, Q23.3, I35.1, I35, Q23.3, I05.1, I37.1, I37, I35.1, I05.8, I06.0, I34.1 |
| HIV | 19030005, 713722001, 86406008, 397763006, 713572001, 713880000, 103412005, 103413000, 713490002, 103418009, 44274007, 103414006, 103408004, 713897006, 186708007, 103411003, 713484001, 442537007, 186705005 | 42 | B22.0, B20.6, B22, B21.7, B20.4, B22.1, B22.2, B20.8, B21, B20.2, B20.3, B21.9, B21.0, B21.1, B21.3, B24, B22.7, B21.2, B20.1, B20.0, B20.9, B20.5, B21.8, B20.7, B20 |
| Myelodysplastic Syndrome | 109998009, 189509003, 398623004, 277597005, 307651005, 109995007, 109994006 | 238.73, 238.72, 238.74, 238.75, 238.76, 238.71 | D46.0, D46.9, D46.5, D46.6, D46.0, D46.2, D46.1, D46.4, D46.9, D46, D46.7, D46 |
| Bone Marrow Transplant | 445757003, 23719005, 2631000119108, 446253009, 153351000119102, 30438009, 19944001  46280001, 737300001, 234336002 | 41.04, 41.01, 41.05, 41.07, 41.08, 41.03, 41.3, 41, V42.82, 41.09, 41.02, V42.81, 41.06, 41.92 | Z94.81, Z94.84 |
| Central Venous Catheter | 736152001, 473083009, 310643001 | 999.31, 999.32, 999.33 | T80.211A, T80.218A, T80.212A, T80.219A |
| Solid Organ Transplant | 737295003, 739024006, 737297006, 737296002, 698367001, 698362007, 313039003 | V42.0, V42.7,  V42.1, V42.6, V42.84, V42.83 | Z94.0, Z94.4, Z94.1, Z94.2,  Z94.82,  Z94.83, Z94.3 |
| Peptic Ulcer with no GI Bleeding | 35517004, 76796008, 40214005, 5492000, 2783007, 4269005, 196698007, 56776001, 73481001, 37442009, 196690000, 128287004, 196683005, 196682000, 51868009, 397825006, 13200003 | 533.9, 534.9,  531.9, 534.7, 532.7, 531.7,  533.7, 532.9 | K27.9, K26.9, K28.9, K25.9, K27.7, K28.7, K26.7, K25.7 |
| Congestive Heart Failure | 42343007, 194779001, 85232009, 83521008,  276514007, 194781004, 85898001, 5148006, 8501000119104, 46113002, 399020009, 23685000,  415295002, 84114007, 194849004 | *** | I43.2, I42.7,  I50.0, I43.1,  I13.0, I25.5,  I42.0, I42.9,  I09.9, I42.6,  I50.1, I50, I42.5, I13.2, I11.0, I50.9 |
| Other Neurological Conditions | 230232005, 5262007, 192979009, 10394003, 91175000, 58756001, 763597000, 230414008, 46808003, 80690008, 230456007, 39912006, 19598007, 36803009, 24700007, 47000000, 192874000, 49692006, 60576007, 230292008, 230380005, 72986009, 238153009, 65120008, 64383006, 230296006, 230227009, 386766007, 41497008, 719848005, 278509004, 118940003,  77659000, 230460005, 230381009, 81308009,  230390002, 230304003, 362975008, 154981003,  91502009, 6807001, 418143002, 87486003, 271700006, 128613002, 19972008, 4223005, 407675009, 117891000119100, 23732000, 13973009,  267679005, 241006, 7033004, 37340000, 50866000, 389098007, 106169008, 85672005, 15244003, 25044007, 193022009, 32798002, 85102008, 6118003, 230379007, 267574006, 84757009, 723437000, 49049000, 135761000119101 | 341.8, 340,  334.4, 345.6,  334.3, 348.3,  345.1, 334.2,  334.8, 345.5,  345.4, 341.1,  333.92, 784.3,  345.7, 335.2,  345.3, 345.9,  345.8, 334.9,  335.8, 333.4,  335.9, 341.9,  334, 348.1,  341, 345, 335.1, 335,  332.1, 780.3,  345.2, 334.1,  331.9, 336.2,  333.5, 332 | G40.4, G12,  G12.1, G20,  G40, G36,  G12.9, G40.2, G40.1, G40.8, G31.2, G21.1, G36.8, G37,  G11.4, R56,  G13, G11.2,  G11, G10,  G32.0, G37.9, G12.2, G21.2, G12.0, G32,  G40.5, R56.0, G37.8, G93.1, G21.9, G21.3, G21.4, G11.0, G13.8, G40.3, G21,  G36.1, G32.8, G31.8, G41.8, G37.1, G13.2, G40.6, G37.4, G37.0, G13.1, G40.0, G11.1, G41.2, G41.0, G13.0, G37.2, G36.0, R56.8, G41,  G37.3, G41.9, G31.9, G41.1, G40.9, G21.8, G25.4, G93.4, R47.0, G11.8, G12.8, G36.9, G35,  G37.5, G25.5, G11.9, G40.7, G11.3, G21.0, G22 |
| Obesity | 238131007, 162864005 190965006, 415530009 414916001, 190966007 | 278 | E66.9, E66.0, E66,  E66.8, E66.2, E66.1 |
| Blood Loss Anemia | 724556004, 413533008, 191127009, 413532003, 267530009, 42626004, 413531005 | 280.0 | D50.0 |
| Weight Loss | 139091004, 238108007, 161834000, 74257000, 77091003, 267158006, 238109004, 58262005, 267024001, 190602008, 190603003, 272588001, 77702009, 29740003, 89362005, 238107002, 360549009 | 262, 799.4, 261, 783.2, 263.8, 263, 263.2, 260, 263.9, 263.1 | E46, E44.0, E45, E41, R63.4, E40, E44, R64, E42, E44.1, E43 |
| Psychoses | 64905009, 79866005, 38368003, 29599000, 31373002, 268624000, 48500005, 76566000, 191559008, 191564007, 44376007, 88975006, 26025008, 712850003, 712824002, 191563001, 59617007, 231437006, 84760002, 31658008, 765176007, 270901009, 12939007, 70814008, 26472000, 27387000, 191562006, 35252006, 191542003, 231487004, 231485007, 30336007, 5464005, 61831009, 51133006, 58214004, 41832009, 278853003, 191570001, 16990005, 191554003, 69322001, 35218008, 71103003, 428703001, 14291003, 191586008, 191676002, 191574005, 191527001, 191571002, 191668004, 191555002, 191531007, 191623007, 271428004, 191565008, 191572009, 231489001, 83746006, 191525009, 4926007, 191547009, 191561004, 1089681000000100, 191678001, 111483008, 191569002, 68890003, 68995007, 63181006, 111482003, 111484002, 191548004, 191680007, 42868002, 191590005, 191526005 | 295.45, 295.92,  295.95, 298.1,  295.85, 295.21,  297.9, 293.8,  297.8, 295.43,  295.75, 295.82,  295.42, 295.13,  295.71, 295.02,  295.73, 295.9,  295.8, 295.3,  295.24, 295.65,  295.51, 295.61,  295.52, 295.93,  295.6, 295.64,  295, 296.44,  295.14, 295.84,  295.23, 295.33,  298.9, 295.35,  297.3, 295.01,  296.04, 298.2,  295.12, 297.2,  297, 295.53, 295.44, 297.1,  295.41, 295.34,  295.94, 295.81,  295.25, 298.8,  295.11, 298.4,  298, 295.63,  295.55, 295.22,  295.74, 296.14,  295.32, 295.15,  295.5, 295.04,  295.62, 295.2,  296.54, 295.1,  295.03, 295.72,  295.91, 298.3,  295.05, 295.4,  295.31, 295.54,  295.7, 295.83 | F30.2, F25.8,  F20.1, F20.9,  F20.0, F28,  F25.9, F23,  F25, F20.5,  F23.8, F29,  F23.1, F22.9,  F22, F20.4,  F23.9, F20.2,  F25.2, F20,  F31.5, F20.8,  F25.0, F31.2,  F20.3, F22.8,  F23.3, F25.1,  F23.2, F24,  F20.6, F23.0, F22.0 |
| Pulmonary Circulation Disorder | 87433001, 25897000, 78723001, 90623003, 48347002, 32398004, 86638007, 195977004, 846635004, 14700006, 196049002, 37471005, 87837008, 62371005, 274096000, 22607003, 195967001, 17385007, 73144008, 68328006, 52333004, 61937009,  17996008, 29422001, 67242002, 13645005,  196017002, 45145000, 8247009, 196001008,  13394002, 12295008, 805002, 426853005, 195951007,  733858005, 51277007, 85761009, 266361008,  13151001, 196053000, 424643009, 74417001,  389145006, 63480004, 195953005, 4981000, 74015002, 286964001, 40122008, 18690003, 57686001, 69339004, 50043002 | 505, 503, 496, 491, 494, 495, 493.2, 504, 416.8, 493.1, 506.4, 501, 502, 490, 492.8, 416.9, 508.8, 508.1, 493, 500, 492 | J66.1, J61,  J43, J63.0,  J67.5, J63,  J67.8, J67.6,  J62.0, J43.8,  J41.0, J67.1,  J42, J45.8,  J66.0, J70.1,  J67.4, J47,  J66.2, J41.8,  J67, J70.3,  J67.9, J44.0,  J44.9, J66,  J44, J46, J45.1, J66.8,  J63.1, J41,  J67.2, J44.8,  J62, J41.1,  J44.1, J63.5,  J43.0, I27.8,  J64, J68.4,  J40, J43.9,  J65, J67.3,  J63.3, J62.8,  J43.1, J67.0,  J45, J67.7, J63.8, J43.2, J45.9, J45.0, I27.9, J63.2, J60, J63.4 |
| Hypertension | 59621000, 1201005, 194760004, 38341003, 161501007, 78975002, 60899001, 123799005, 8501000119104, 89242004, 31992008, 104931000119100, 65443008, 46113002, 64715009, 73410007, 194781004, 49220004, 86234004, 5148006, 28119000, 38481006, 193003, 194783001, 194788005, 194779001, 194785008, 194780003 | 401.9, 401, 401.1, 403.1, 404, 402, 403, 405.11, 405.01, 405.1, 405 | I13, I11.9, I13.1, I13.9,  I12.9, I15.2,  I15.1, I15.8,  I15.9, I12, I12.0, I15.0,  I15, I11, I13.2, I13.0,  I11.0 |
| Hypothyroidism | 26692000, 27059002, 237567008, 367161000119106,  21263006, 190304001, 271949009, 237566004, 43153006, 237555006, 428165003, 216693007, 237527007, 82598004, 237558008, 40930008, 3716002, 237571006, 83986005, 367631000119105, 10809101000119100, 286910004, 191038007,  190268003, 237565000, 217710005, 278503003,  111566002, 716338001, 405629002, 237528002, 237515009, 64491003, 37429009, 14304000, 237562002, 190279008, 54823002, 2917005 | 244.1, 246.1,  246.8, 244.9,  244, 240.9, 243 | E00.9, E03.8,  E00.0, E03.2,  E89.0, E03.0,  E02, E00.1, E01.1, E00, E03.4, E03.3,  E03.9, E03,  E00.2, E01.2,  E01, E03.1,  E03.5, E01.8,  E01.0 |
| Coagulopathy | 267532001, 75331009, 234467004, 32273002, 362970003, 28293008, 302215000, 49762007, 26843008, 154826009, 267535004, 128105004, 86075001, 191287000, 41788008, 64779008, 25904003, 267534000, 73162004, 67406007, 785308008, 439698008 | 286.2, 287.31, 287.1, 287.32, 286.9, 286.5, 287.5, 286.52, 286.1, 286.4,  287.49, 287.3,  287.39, 286.53,  287.33, 286.3,  286, 286.59, 286.6, 287.41, 286.7, 287.4 | D67, D68.8,  D68.6, D65,  D68.3, D68.2,  D68.9, D68.0,  D69.1, D69.3,  D68.5, D68,  D66, D69.6,  D68.4, D69.4,  D68.1, D69.5 |
| Diabetes | 14735008, 420996007, 190395005, 111552007,  421750000, 422126006, 420422005, 190373006,  12811000119100, 313436004, 190406000,  420270002, 190412005, 420662003, 44054006, 313435000, 73211009, 422166005, 421256007,  74627003, 190411003, 441628001, 75524006,  420279001, 420683009, 422088007, 190407009,  46635009, 422099009, 421893009, 25093002,  371054002, 421468001, 190366001, 190385003,  420414003, 422014003, 190388001, 371056000,  421326000, 739681000, 190365002, 190387006,  190386002, 420868002, 190363009, 422228004, 421895002, 127013003, 190410002, 421365002, 190364003 | 250.1, 250.2,  250.3, 250, 250.6, 250.9, 250.8, 250.7, 250.4, 250.5 | E12.0, E11.1,  E11.0, E14.0,  E14.9, E10.1,  E13.0, E13.1,  E10.0, E11.9,  E10.9, E13.9,  E14.1, E12.1,  E12.9, E14.5,  E13.4, E12.2,  E13.7, E12.5,  E10.3, E14.8,  E11.5, E10.7,  E11.7, E14.2,  E11.2, E13.8,  E14.3, E11.4,  E11.3, E13.6,  E12.4, E14.6,  E10.5, E13.2,  E12.8, E13.5,  E14.4, E12.3,  E12.6, E10.4,  E10.2, E13.3,  E10.6, E11.6,  E12.7, E14.7,  E10.8, E11.8 |
| Paralysis | 192966000, 11538006, 29426003, 54099005, 58193001, 1593000, 192967009, 192964002,  56409008, 43486001, 609557004, 192970008,  50582007, 39912006, 275468009, 44695005, 79633009, 304737009, 41764006, 80935004, 22881000119100, 80420005, 60389000, 128188000, 86022000, 29188005, 198030008, 714279000, 192965001, 813921000000104 | 344, 342.9, 343.2, 344.1,  344.9, 343.1,  344.6, 343.3,  342.1, 334.1,  344.5, 344.2,  344.3, 342, 344.4, 343.4, 343.8, 343.9, 343 | G80.2, G82.1,  G81.9, G83.3,  G83.0, G83.2,  G80.1, G83.9,  G04.1, G82,  G82.5, G83.4,  G82.3, G83.1,  G82.4, G81.0, G81, G82.0,  G11.4, G81.1,  G82.2 |
| Fistula | 197153005, 111356006, 786878009, 58103005, 271023002, 95435007, 15165002, 50477003, 307239003, 53206008, 40046003, 204654008, 28626004, 204268008, 204659003, 197155003, 111751003, 235671002, 38851006 | 569.69, 569.81 | K94.19, K63.2 |
| Liver Disease | 72836002, 444918006, 17709002, 50325005,  371067004, 186639003, 161535005, 57339008,  34742003, 235880004, 62484002, 195474004,  41889008, 14223005, 59927004, 408335007,  235869004, 1082601000119100, 197284004, 41309000, 1082621000119100, 195476002, 28670008,  12368000, 235901004, 79720007, 195475003,  420054005, 442685003, 111891008, 197321007,  235899008, 435101000124104, 1085091000119100,  31712002, 235881000, 76783007, 424340000, 235886005, 1761006, 19943007, 235856003, 186624004, 128302006, 266468003 | 70.44, 70.32, 572.8, 70.22, 571.41, 571.5,  573.5, 571.49,  571.42, 571.2,  456.1, 70.23,  456.2, 571.3,  571.4, 572.3, 571.6, 456, 70.33, 571, 571.9, 571.8, 456.21, 70.54 | K70.41, K72.90, K75.81, I85.11, K72.10, K70.30,  K70.40, I85.10, K74.69, K72.11,  I85.00,  K76.89,  I85.01,  K72.91,  K70.31, K74.60,  B18.1, K76.9, K74.1, B18.0,  K74.4, K70.0,  K74.3, K76.6,  K73.8, K73.1,  K73.9, K70.9,  K74.2, K76.0,  K74.0, B18.2,  K75.4, K74.5,  K70.2, K73.2, K73.0 |
| Metastatic Cancer | 94225005, 813671000000107, 127254000, 94348003, 269473008, 94396003, 94347008, 94297009, 94391008, 94398002, 94351005, 94395004, 94442001, 94313005, 188462001, 127267002, 94350006, 127261001, 94649002, 94360002, 94515004, 94628003, 94365007, 94580002, 188445006, 94493005, 274088005, 94161006, 94663008, 94455000, 285645000, 127274007, 127245003, 94381002, 94409002, 363346000, 94326009, 94579000, 94222008, 94392001, 127250009, 254289008, 127232002 | 196.5, 197.8,  198.5, 196.9,  196.8, 196.2,  196.1, 196.6,  199.1, 197.3,  198.2, 196.3,  198.3, 197.5,  197.4, 197,  198.7, 197.6,  198.4, 198.1,  196, 198.8, 197.2, 197.7, 198.81, 199.2, 199, 198.82, 197.1, 198.89, 198.6, 198 | C77.0, C77.9,  C77.8, C79.4,  C78.3, C77.4,  C78, C78.2,  C78.4, C77.5,  C79.1, C77.3,  C78.7, C79.8,  C77.1, C78.1,  C79.7, C77,  C78.6, C79.2,  C79.3, C77.2, C78.8, C78.5,  C80, C80.0,  C79.0, C79.5,  C79, C79.6,  C79.9, C78.0,  C80.9 |
| Arthritis | 10317009, 410795001, 62918002, 371082009, 65323003, 317931000119101, 85551004, 201051000,  161567008, 75053002, 239792003, 89155008,  195363007, 55146009, 417373000, 74391003,  359789008, 33719002, 195353004, 410797009, 33760009, 22784002, 57160007, 75822003, 239796000, 69896004, 201436003, 310701003, 400054000, 1961000, 9631008 105969002, 201799006, 84801008, 398726004, 239920006, 50442003, 31848007, 239791005, 319841000119107, 239887007, 23685000, 201796004, 239805001 | 714.31, 720.9, 446.7, 725, 714.2, 714.1, 446, 720.8, 710.9, 711.2,  720.81, 446.21,  720.1, 720.89, 720, 714.89, 714.81, 714.9, 714.4, 728.5, 714.32, 714,  710, 714.33,  446.2, 719.3,  714.8, 720.2, 714.3 | M34.0, M45.0, M35.3, M32,  M33.0, M30,  L94.3, M06.1,  M08.9, M05.0, M34.8, L94.0,  M35.7, M06.3, M06.2, M30.0, M05.3, M05.1, M30.8, M08.8, M32.9, M05.2, M08.0, M30.2, M35.9, M08.3, M46.9, M33.9, M34.2, M12.3, M08,  M32.0, M08.2, M05.9, M35.2, M06,  M32.8, M08.4, M06.8, M08.1, M05.8, M30.1, M06.4, M12.0, M34.1, M46.8, M33.2, M45,  M06.0, M46.1, M06.9 |
| Deficiency Anemia | 70241007, 66612000, 191161006, 267517008, 49472006, 191142007, 10619002, 267513007, 87522002, 371315009, 53165003, 237934001, 84027009, 267518003, 85649008, 85746008 | 280.8, 281.1,  281, 281.4, 281.2, 281.8,  280.1, 281.3, 280.9, 281.9 | D52.8, D53.2,  D51.3, D51.2,  D52.9, D52.0,  D52.1, D53,  D51.8, D50.9,  D53.1, D53.8,  D53.9, D51.1,  D50.8, D51.9, D51, D52, D51.0, D53.0 |
| Lymphoma | 847741000000106, 421418009, 414166008, 55150002,  1929004, 77430005, 404134006, 21964009, 372087000, 269475001, 39795003, 109988003,  109968002, 445269007, 109989006, 369767007,  308121000, 118615008, 188498009, 118605002,  269476000, 444910004, 188487008, 277637000,  443487006, 109980005, 109977009, 109958007,  277613000, 109965004, 129000002, 118608000,  118599009, 68979007, 109972003, 404143002,  109966003, 109979007, 448212009, 110459008,  448354009, 3172003, 118612006, 190818004,  404148006, 118609008, 447100004, 449220000,  46732000, 64575004, 103686008, 369766003,  118614007, 118601006, 109985000, 302841002,  14537002, 128803008, 112687003, 415111003,  118618005, 118607005, 118613001, 118610003,  118617000, 109970006, 188718006, 449176003,  109971005, 373168002, 473068004, 77381001, 307649006, 118611004, 446643000, 118600007, 109962001 | 200.1, 202.2,  202.1, 200.3,  200.7, 202.8,  238.6, 200, 200.8, 200.6, 202.9, 202.5,  200.2, 202.4, 200.5, 202.6,  202.3, 201, 203, 202.7, 202, 200.4 | C84.6, C96.2,  C82.4, C84.4,  C83.1, C84.7,  C84, C82.2,  C90.2, C96.5,  C85.2, C96.6,  C84.1, C88,  C84.9, C96.4,  C83.0, C83.9,  C96, C85.7,  C88.7, C83,  C84.8, C81.3,  C82.3, C83.5,  C81.4, C82,  C85.1, C96.7,  C88.9, C88.4,  C81.9, C82.1,  C96.8, C81.2,  C84.5, C82.0,  C88.0, C82.5,  C85, C83.8,  C82.6, C90.0,  C83.7, C88.2,  C82.9, C81,  C83.3, C96.9,  C82.7, C88.3,  C81.7, C81.1,  C81.0, C85.9,  C84.0, C96.0 |
| Pancreatitis | 197456007, 47367009, 235942001, 235944000,  235943006, 111374002, 197461009, 235952002,  235960001, 197457003, 235941008, 15528006, 4399003, 75694006 | 577 | K85.22, K86.81, K85.31, K85.12, K85.82, K85.92, K85.80, K85.81, K85.20, K85.02, K85.10, K85.90, K85.32, K85.00, K85.91, K85.01, K85.21, K85.11, K85.30, K86.0, K86.3 |
| History of Cardiac Surgery | 29819009, 359597003, 90487008, 39724006, 161625008, 14323007, 10190003 | 36.12, 36.13, 36.11, 36.2, 36.17, 36.16, 36.14, 36.1, 36.15, 36.19 | 02124J3, 02134AW, 212488, 21008, 021348F, 02100K3, 02100ZF, 021308C, 210489, 021308W, 021009W, 02104ZF, 02104KW, 021048W, 021048C, 02130J9, 021248W, 021248F, 212489, 02104AC, 021149W, 02134A9, 02104KC, 02130JC, 02100KC,  021349F,  02130AW,  211498,  212498,  02124K8,  02100Z9,  02130KW,  02124KC,  02124A,  021249W,  02134Z9,  02130JW,  02134K9,  02104A8,  02104ZC,  02134A8,  02104Z9,  02130J3,  021108F,  02130A,  021009F,  02104J3,  02110AW,  02130Z,  021008C,  02134D,  02124K,  02104A3,  02130ZC,  02130K9,  02124J9,  021249C,  02134J8,  02134AF,  02100J,  02130Z3,  021349W,  02124D4,  02124KW, 02124AW,  02100A,  02100KF,  02124A3,  02130KF,  02134D4,  02130A3,  021008W,  02124ZC,  02104J8,  021048F,  02130Z9,  02104J,  02100A8,  02104K,  02124JF,  021049C,  02100K,  02134J3,  02130AF,  02100Z8,  02104K9,  02134ZF,  02124J8,  02134Z8,  02130Z8,  02100K8,  02134K8,  02124Z8,  02100A9,  02124J,  02130K3,  02134Z3,  02124Z9,  02104Z8,  02134AC, 02130AC,  021149F,  02134K3,  02100AF,  021208F,  02134KW,  02104Z,  02100JC,  02124A8,  02124D,  021249F,  02100KW,  02130J,  021009C,  02124JW,  02134KF,  02110AF,  02130K,  02100AC,  021308F,  02110A,  021149C,  021348W,  02134JW,  02104JW,  02110A3,  02124A9,  021008F,  02130A8,  02124K3,  02134J9,  02104D,  02134J,  02130K8,  02100J8,  021348C,  02104D4,  02100AW,  02100JF,  02134A3,  021049W,  02104KF,  02104A,  02104Z3,  02104J9,  02100J3,  02104K3,  02134ZC,  02100JW,  02124JC,  021309C,  02100J9,  02100Z3,  02100ZC,  02104AF,  02104JF,  02104K8,  02104AW,  02104JC,  02134A,  02134Z,  02130JF,  02130A9,  02104A9,  02100K9,  02124Z3,  02124ZF,  021349C,  02130ZF,  02134K,  02124AF,  02110AC,  02124Z,  021248C,  02134KC,  021049F,  02134JF,  02134JC,  02124K9,  02124AC,  02110A8,  02130J8,  02110A9,  02100A3,  02100Z,  02124KF,  021309W,  02130KC,  021309F |

***Classification not used.

Appendix Table 3. CAIFI associated laboratory results used to assign cases

| CAIFI | OMOP Concepts | Standardized Codes | Type of Test |
| --- | --- | --- | --- |
| *Candida* | 36203582, 42868513, 36203228, 2213108, 36203584, 3023368 | *LOINC:* 85782-1, 70024-5, 85781-3, 85784-7, 600-7*  *CPT4:* 87480 | 1. *Candida* spp. isolated in blood. |
| *Aspergillus* | 3046760, 3035826, 3045331, 3036424, 40765204 | *LOINC:* 44357-2, 35383-9, 62467-6, 35671-7, 44099-0 | 1. Galactomannan in Serum > 0.7 OD 2. Galactomanna in BAL > 0.8 OD 3. Galactomannan in CSF > 0.8 OD |
| *Cryptococcus* | 3027604, 3010749, 3024255, 3001363, 3004834, 3007792, 3039830, 42869378, 3022450, 3023174, 3023368, 3027446, 3050716, 3051966, 4284105, 21493347 | *LOINC:* 9820-2, 9819-4, 29533-7, 29903-2, 40768-4, 31788-3, 70910-5, 29896-8, 11473-6, 46964-3, 600-7*, 49098-7, 31790-9, 30324-8, 82181-9  *SNOMED:* 67168003 | 1. Cryptococcal antigen in serum and CSF. 2. *Cryptococcus* isolated in blood culture. |
| *Coccidioides* | 3006465, 3021763, 3000779, 3005750, 3035855, 3043731, 3049784, 3049822, 40765196 | *LOINC:* 27009-0, 5095-5, 13947-7, 22209-1, 48392-5, 33380-7, 7826-1, 48588-8, 62459-3 | 1. *Coccidioides* antigen. 2. *Coccidioides* antibody. |

* LOINC code 600-7 codes to bacteria identified in blood culture, however the recorded species for results in this code were either *Candida albicans, Candida glabrata*.

Loinc codes can be referenced at <https://loinc.org/>. CPT can be found here: <https://www.ama-assn.org/practice-management/cpt/cpt-overview-and-code-approval>. Additional details about OMOP standardized vocabularies can be found at <https://www.ohdsi.org/web/wiki/doku.php?id=documentation:vocabulary:sidebar>, while OMOP concepts can be found at <https://athena.ohdsi.org/search-terms/start>, and additional details about OMOP are documented by OHDSI here: <http://ohdsi.github.io/CommonDataModel/index.html>.

Appendix Table 4. ICD-9, ICD-10, SNOMED-CT clinical diagnostic codes of invasive fungal infections

| Concept | ICD-9, ICD-10,SNOMED-CT code |
| --- | --- |
| Aspergillosis | *ICD-9:* 117.3  *ICD-10:* B44  *SNOMED-CT:* 65553006 |
| Pulmonary aspergillosis | *SNOMED-CT:* 6042001 |
| Invasive pulmonary aspergillosis | *ICD-10:* B44.0  *SNOMED-CT:* 3214003 |
| Pneumonia in aspergillosis | *ICD-9:* 484.6  *SNOMED-CT:* 111900000 |
| Disseminated aspergillosis | *ICD-10:* B44.7  *SNOMED-CT:* 7671008 |
| Invasive aspergillosis | *SNOMED-CT:* 721798004 |
| Infection by *Aspergillus fumigatus* | *SNOMED-CT:* 63764008 |
| Infection by *Aspergillus flavus* | *SNOMED-CT:* 24147008 |
| Sepsis due to Candida | *SNOMED-CT:* 447841007 |
| Candidal endocarditis | *ICD-9:* 112.81  *ICD-10:* B37.6  *SNOMED-CT:* 63553008 |
| Invasive candidiasis | *SNOMED-CT:* 70572005 |
| Candidemia | *SNOMED-CT:* 432261003 |
| Fungemia | *SNOMED-CT:* 240695000, 434532004 |
| Candidal meningitis | *ICD-9:* 112.83  *ICD-10:* B37.5  *SNOMED-CT:* 45021001 |
| Histoplasmosis | *ICD-9:* 115  *ICD-10:* B39  *SNOMED-CT:* 12962009 |
| Cryptococcosis | *ICD-9:* 117.5  *ICD-10:* B45  *SNOMED-CT:* 42386007 |
| Pulmonary coccidioidomycosis | *SNOMED-CT:* 417018008 |
| Cerebral cryptococcosis | *ICD-10:* B45.1  *SNOMED-CT:* 187094001 |
| Blastomycosis | *ICD-9:* 116.0  *ICD-10:* B40  *SNOMED-CT:* 69996000 |
| Infection by *Histoplasma capsulatum* | *ICD-9:* 115.0  *SNOMED-CT:* 76255006 |
| Pulmonary histoplasmosis | *SNOMED-CT:* 187054003 |
| Coccidioidomycosis | *ICD-9:* 114  *ICD-10:* B38  *SNOMED-CT:* 60826002 |
| Pulmonary cryptococcosis | *ICD-10:* B45.0  *SNOMED-CT:* 20953001 |
| Systemic cryptococcosis | *SNOMED-CT:* 187091009 |
| Pulmonary blastomycosis | *SNOMED-CT:* 233616001 |
| Disseminated blastomycosis | *ICD-10:* B40.7  *SNOMED-CT:* 187067000 |
| Disseminated coccidioidomycosis | *ICD-10:* B38.7  *SNOMED-CT:* 85055004 |
| Disseminated *Histoplasma capsulatum* infection | *SNOMED-CT:* 425418002 |
| Acute pulmonary coccidioidomycosis | *ICD-10:* B38.0  *SNOMED-CT:* 187027001 |
| Acute pulmonary histoplasmosis | *SNOMED-CT:* 58524006 |
| Pneumonia due to *Histoplasma capsulatum* | *SNOMED-CT:* 38699009 |
| Chronic pulmonary coccidioidomycosis | *ICD-9:* 114.4  *ICD-10:* B38.1  *SNOMED-CT:* 233615002 |
| Acute pulmonary blastomycosis | *ICD-10:* B40.0  *SNOMED-CT:* 187069002 |
| Chronic pulmonary histoplasmosis | *SNOMED-CT:* 26427008 |
| Pneumonia due to *Histoplasma* | *SNOMED-CT:* 442094008 |
| Cutaneous blastomycosis | *ICD-10:* B40.3  *SNOMED-CT:* 187065008 |
| Meningoencephalitis due to *Blastomyces dermatitidis* | *SNOMED-CT:* 1084741000119103 |

Appendix Table 5. **Incidence of Invasive Fungal Infections Among Intubated Individuals with COVID-19 and Influenza**

| Invasive Fungal Infections (IFI) | Influenza  n (%) | COVID-19  n (%) | p-value |
| --- | --- | --- | --- |
| All IFI | 26/1250 (2.0%) | 1934/68383 (2.8%) | 0.28 |
| Aspergillosis | 14/1250 (1.1%) | 933/68383 (1.3%) | 0.70 |
| Candidemia | 7/1250 (0.5%) | 793/68383 (1.1%) | 0.14 |

Appendix Table 6. **Incidence of Invasive Fungal Infections Among Intubated Individuals with COVID-19 and Respiratory Syncytial Virus (RSV)**

| Invasive Fungal Infections (IFI) | RSV  n (%) | COVID-19  n (%) | p-value |
| --- | --- | --- | --- |
| All IFI | 3/491 (0.61%) | 1934/68383 (2.8%) | 0.01 |
| Aspergillosis | 2/491 (0.41%) | 933/68383 (1.3%) | 0.12 |
| Candidemia | 1/491 (0.20%) | 793/68383 (1.1%) | 0.11 |

Appendix Table 7. **Incidence of Invasive Fungal Infections Among Intubated Individuals (≥96 hours) with COVID-19 and no-COVID-19**

| Invasive Fungal Infections (IFI) | No COVID-19  n (%) | COVID-19  n (%) | p-value |
| --- | --- | --- | --- |
| All IFI | 1406/34602 (4.06%) | 1334/27625 (4.82%) | 0.01 |
| Aspergillosis | 482/34602 (1.39%) | 665/27625 (2.40%) | 0.01 |
| Candidemia | 719/34602 (2.08%) | 570/27625 (2.06%) | 0.92 |

Appendix Table 8. **Incidence of Invasive Fungal Infections Among Intubated Individuals (≥96 hours) with COVID-19 and Influenza**

| Invasive Fungal Infections (IFI) | Influenza  n (%) | COVID-19  n (%) | p-value |
| --- | --- | --- | --- |
| All IFI | 9/246 (3.66%) | 1334/27625 (4.82%) | 0.48 |
| Aspergillosis | 5/246 (2.03%) | 665/27625 (2.40%) | 0.86 |
| Candidemia | 4/246 (1.63%) | 570/27625 (2.06%) | 0.79 |

Appendix Table 9. Incidence of CAIFI stratified by diagnostic criteria (administrative coding and laboratory testing)

|  | Just Administrative Coding | Just Lab | Lab + Administrative Coding |
| --- | --- | --- | --- |
| Aspergillosis | 1.0% | 2.1% | 2.3% |
| *Candida* Invasive Infections | 0.9% | 2.5% | 2.6% |
| Coccidioidomycosis | 0.02% | 3.0% | 1.5% |
| Cryptococcosis | 0.06% | 1.2% | 0.8% |

Appendix Table 10. 30-day and 90-day crude all-cause mortality among patients intubated with COVID-19 by invasive fungal infections

| Invasive Fungal Infection | 30-day crude mortality  n (%) | 90-day crude mortality  n (%) |
| --- | --- | --- |
| Aspergillosis | 415/933 (44.4) | 525/933 (56.2) |
| Candidiasis | 230/793 (29.0) | 375/793 (47.2) |
| Cryptococcosis | 31/90 (34.4) | 46/90 (51.1) |
| Coccidioidomycosis | <20 | 23/63 (36.5) |
| Histoplasmosis | <20 | <20 |
| Mucormycosis | 23/53 (43.3) | 30/53 (56.6) |
| Blastomycosis | <20 | <20 |

*Per N3C policy, no cells with <20 participants should include the exact number.

Appendix Table 11. *Candida* and *Aspergillus* infections association with 90-day mortality by COVID-19 waves

| CAIFI | First Wave  HR [95% CI] | Second Wave  HR [95% CI] | Third Wave  HR [95% CI] |
| --- | --- | --- | --- |
| *Candida* | 1.6 [1.4-1.9] | 1.7 [1.3-2.1] | 1.9 [1.5-2.5] |
| *Aspergillus* | 1.9 [1.6-2.2] | 1.8 [1.5-2.1] | 2.4 [2.0-2.9] |

Appendix Table 12. CAIFI associated with 90-day mortality by clinical and laboratory diagnosis

| CAIFI | Clinical Diagnosis  HR [95% CI] | Laboratory Diagnosis  HR [95% CI] | Laboratory + Clinical Diagnosis  HR [95% CI] |
| --- | --- | --- | --- |
| *Candida* | 1.6 [1.5-1.8] | 1.7 [1.2-2.3] | 1.2 [0.8-1.8] |
| *Aspergillus* | 1.7 [1.5-1.9] | 3.5 [2.7-4.4] | 1.9 [1.5-2.4] |
| *Cryptococcus* | 1.5 [1.1-2.2] | 1.1 [0.7-1.8] | 1.5 [1.0-2.2] |
| *Coccidioides* | 0.8 [0.5-1.4] | 1.1 [0.8-1.7] | 1.1 [0.7-1.8] |

*Infections caused by *Histoplasma*, Mucorales and *Blastomyces* were all diagnosed using ICD-9, ICD-10 or SNOMED-CT codes.

Appendix Table 13. 30-day and 90-day all-cause mortality associated to CAIFI stratified by the administration of systemic antifungals

|  | Received systemic antifungals | | No systemic antifungals | |
| --- | --- | --- | --- | --- |
| CAIFI | 30-day mortality  HR: [95% CI] | 90-day mortality  HR: [95% CI] | 30-day mortality  HR: [95% CI] | 90-day mortality  HR: [95% CI] |
| *Aspergillus* | 1.6 [1.4-1.8] | 1.9 [1.6-2.1] | 2.2 [1.9-2.7] | 2.3 [1.9-2.7] |
| *Candida* | 1.2 [1.1-1.4] | 1.6 [1.5-1.8] | 2.0 [1.7-2.3] | 2.1 [1.9-2.3] |

Appendix Figure 1. Balance of variables after inverse probability weighting among patients with coccidioidomycosis


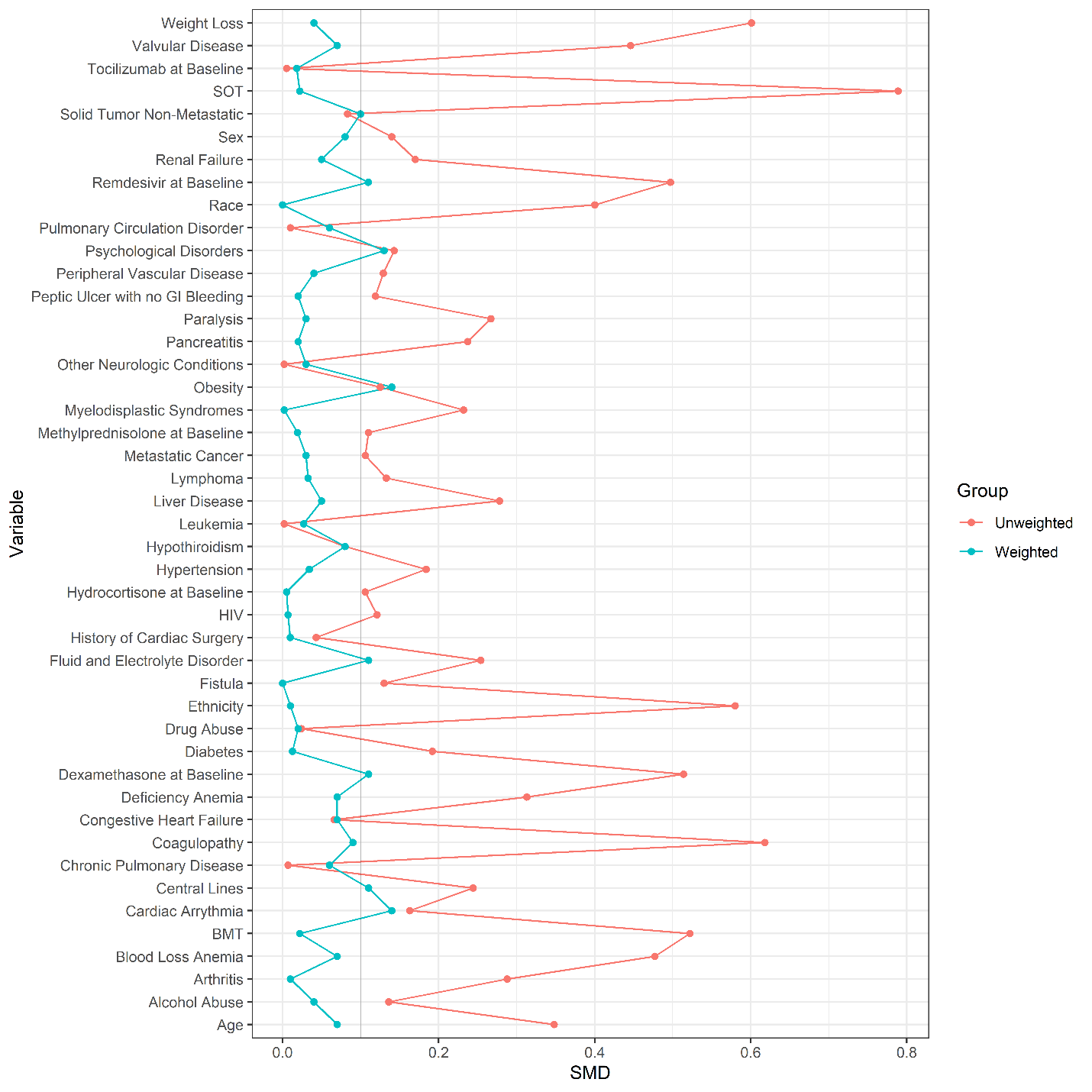


Appendix Figure 2. Balance of variables after inverse probability weighting among patients with blastomycosis


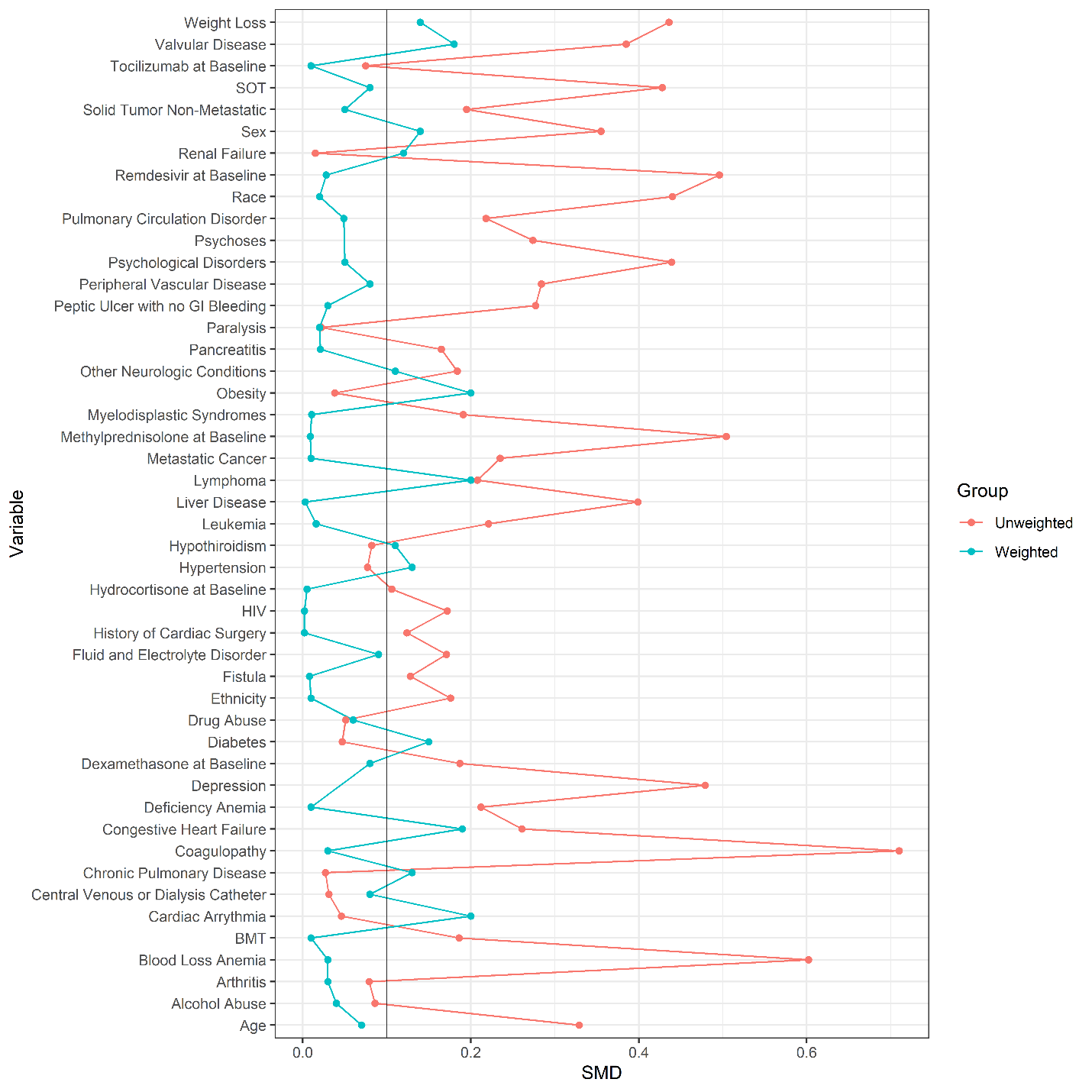


Appendix Figure 3. Balance of variables after inverse probability weighting among patients with mucormycosis


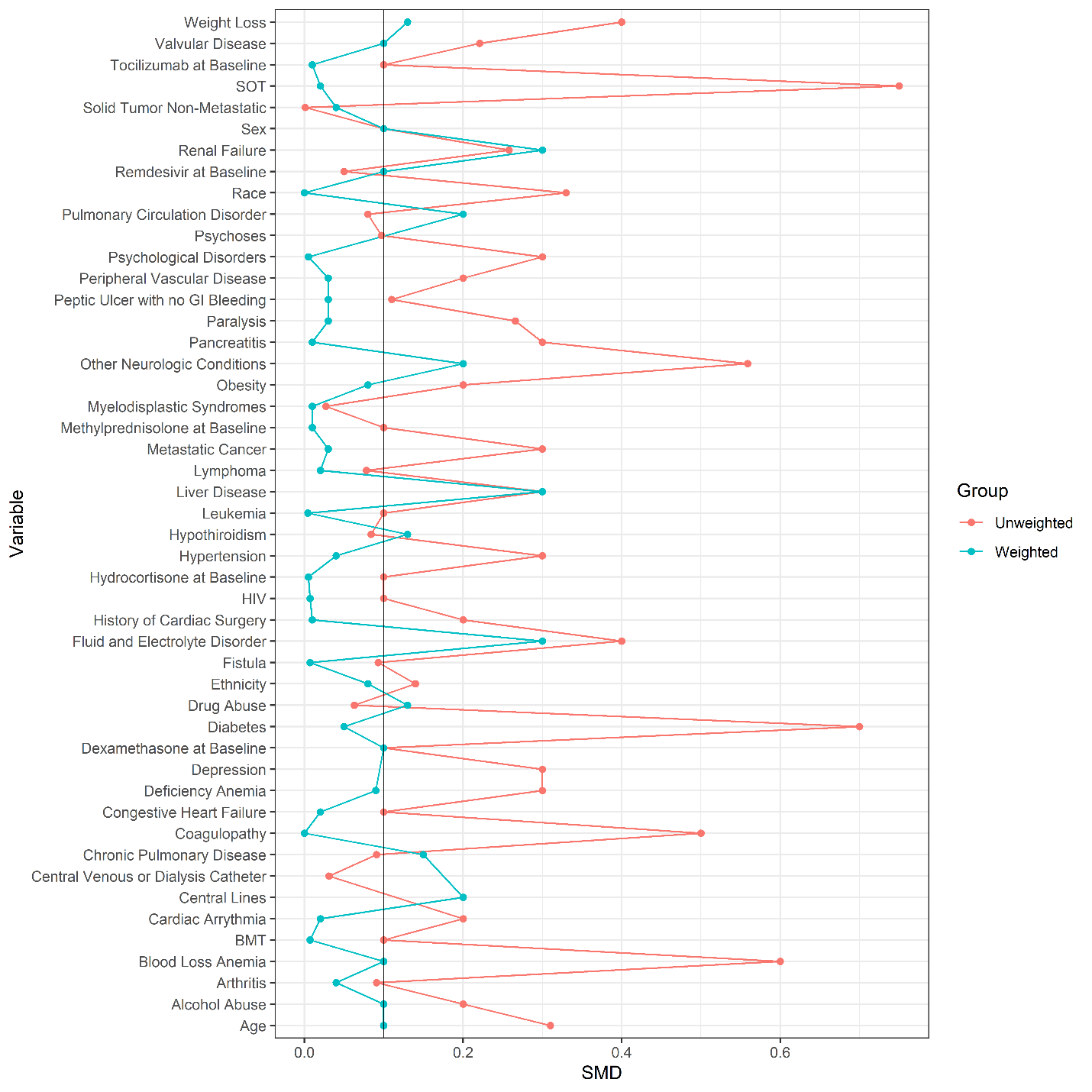


Appendix Figure 4. Balance of variables after inverse probability weighting among patients with histoplasmosis


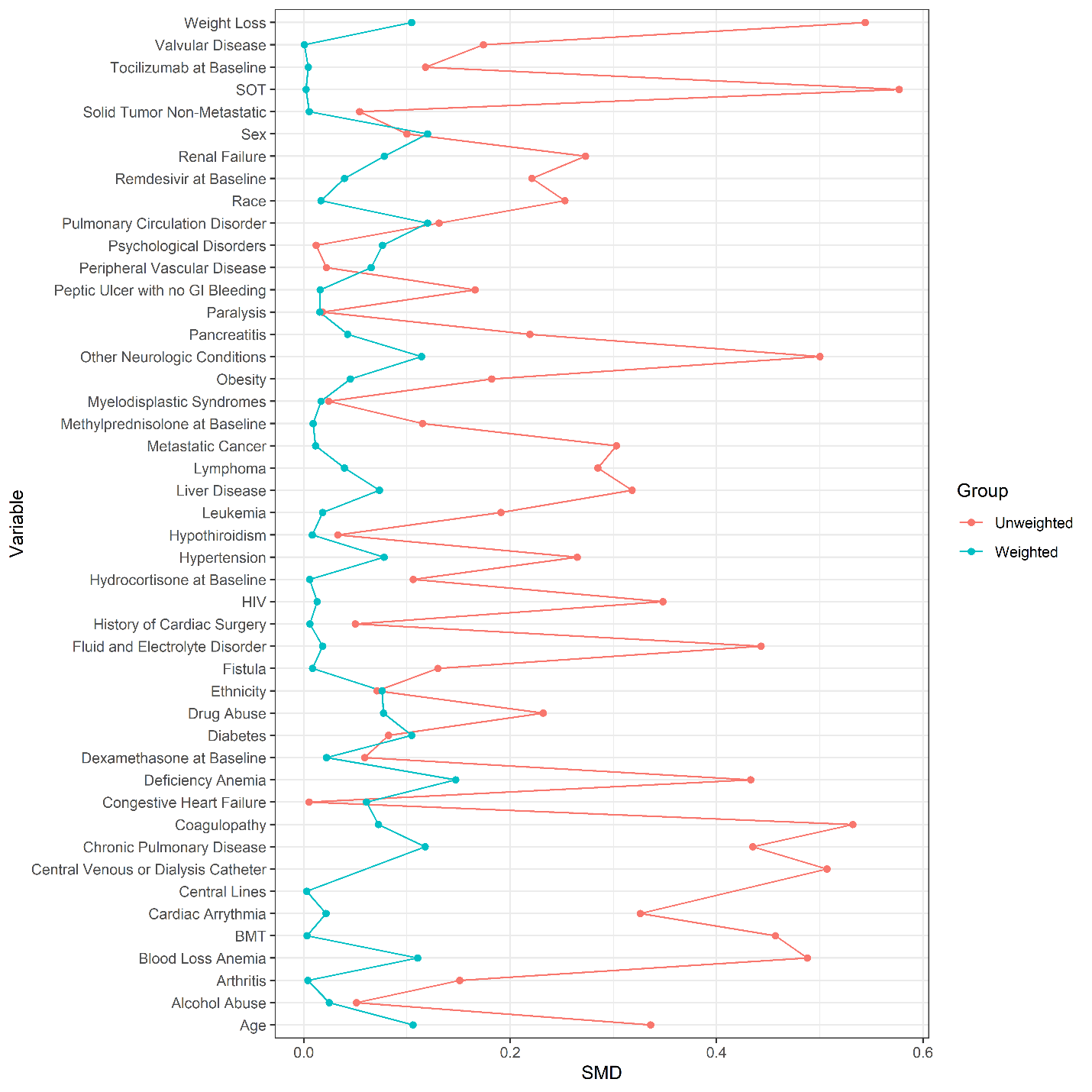


Appendix Figure 5. Balance of variables after inverse probability weighting among patients with cryptococcosis


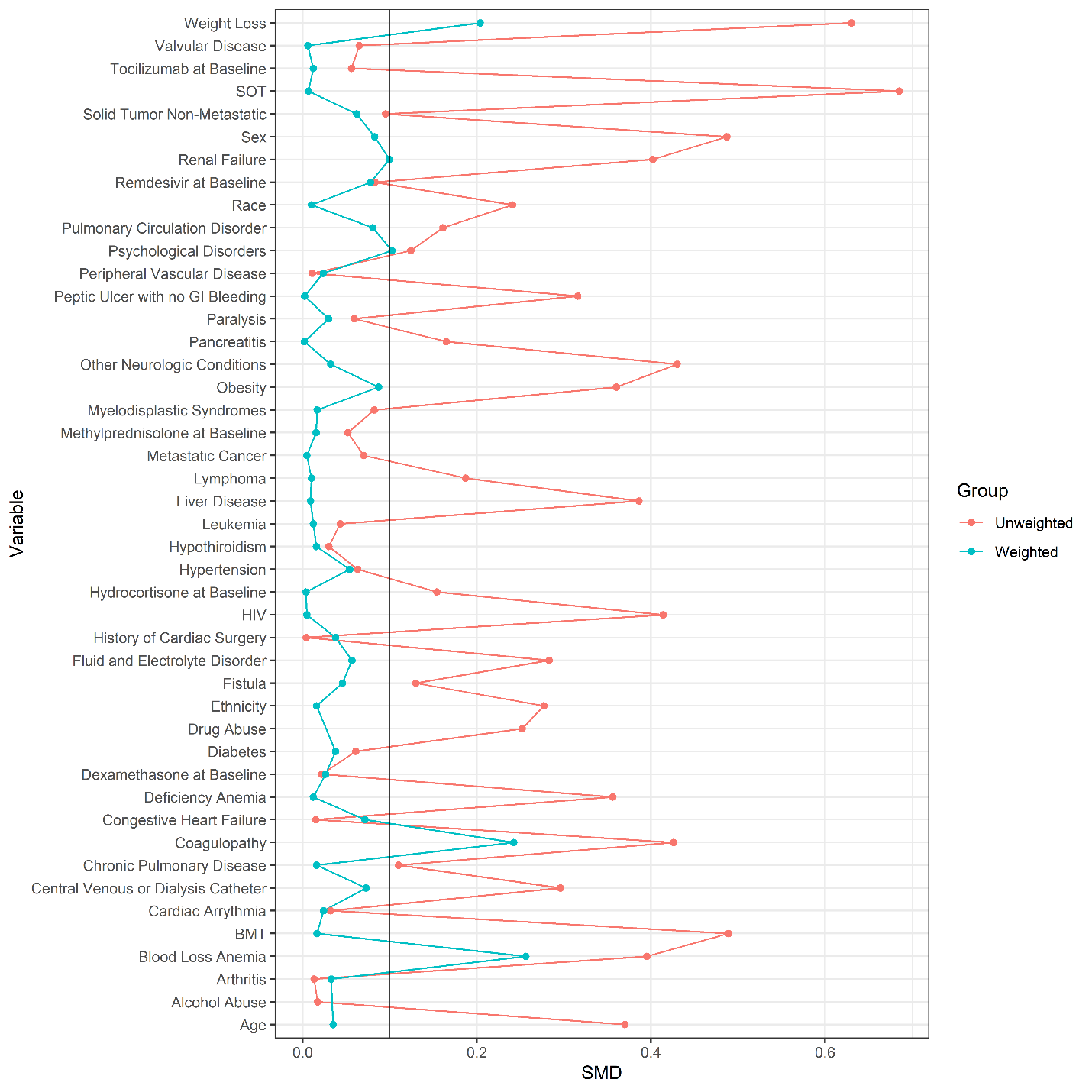


Appendix Figure 6. Balance of variables after inverse probability weighting among patients with aspergillosis


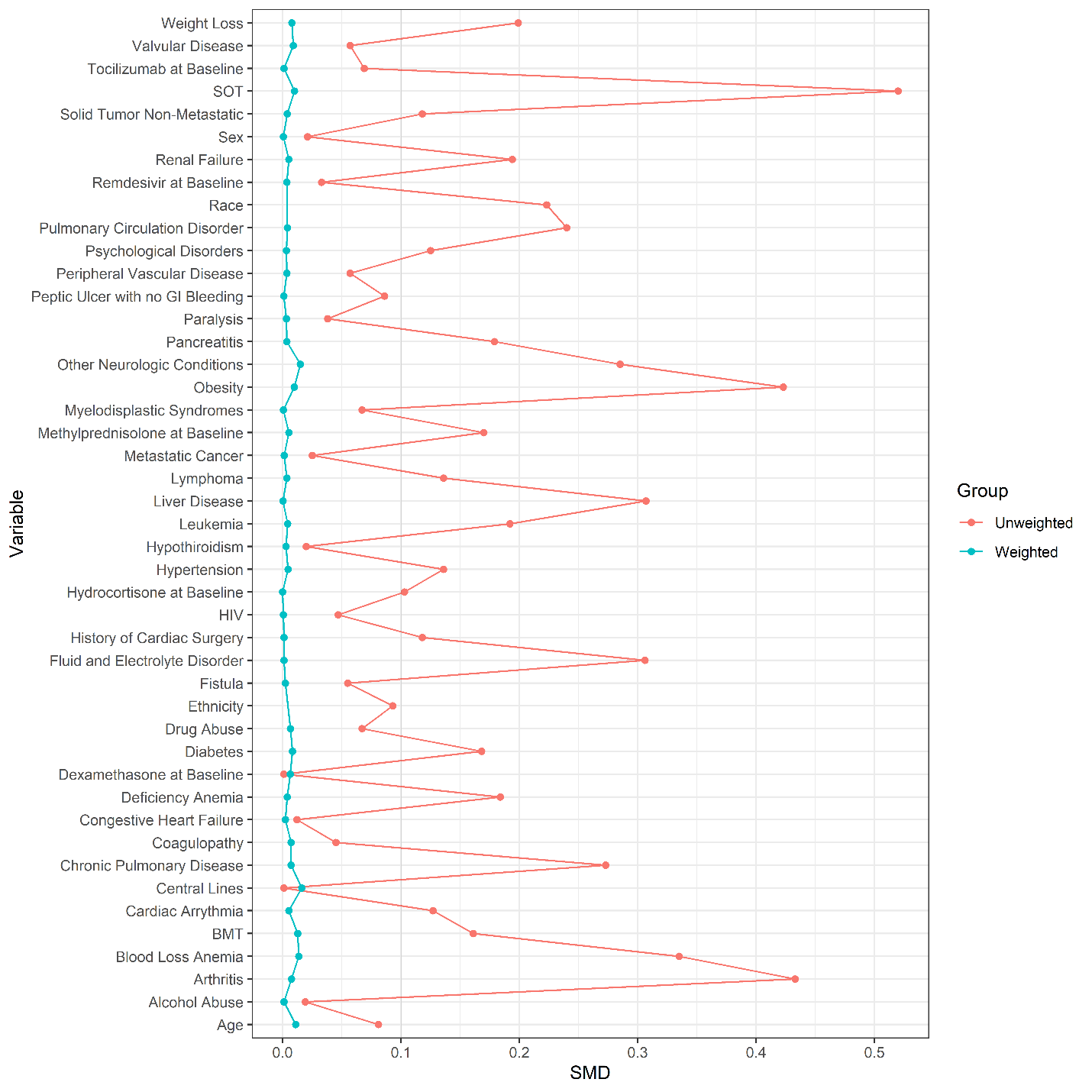


Appendix Figure 7. Balance of variables after inverse probability weighting among patients with candidiasis


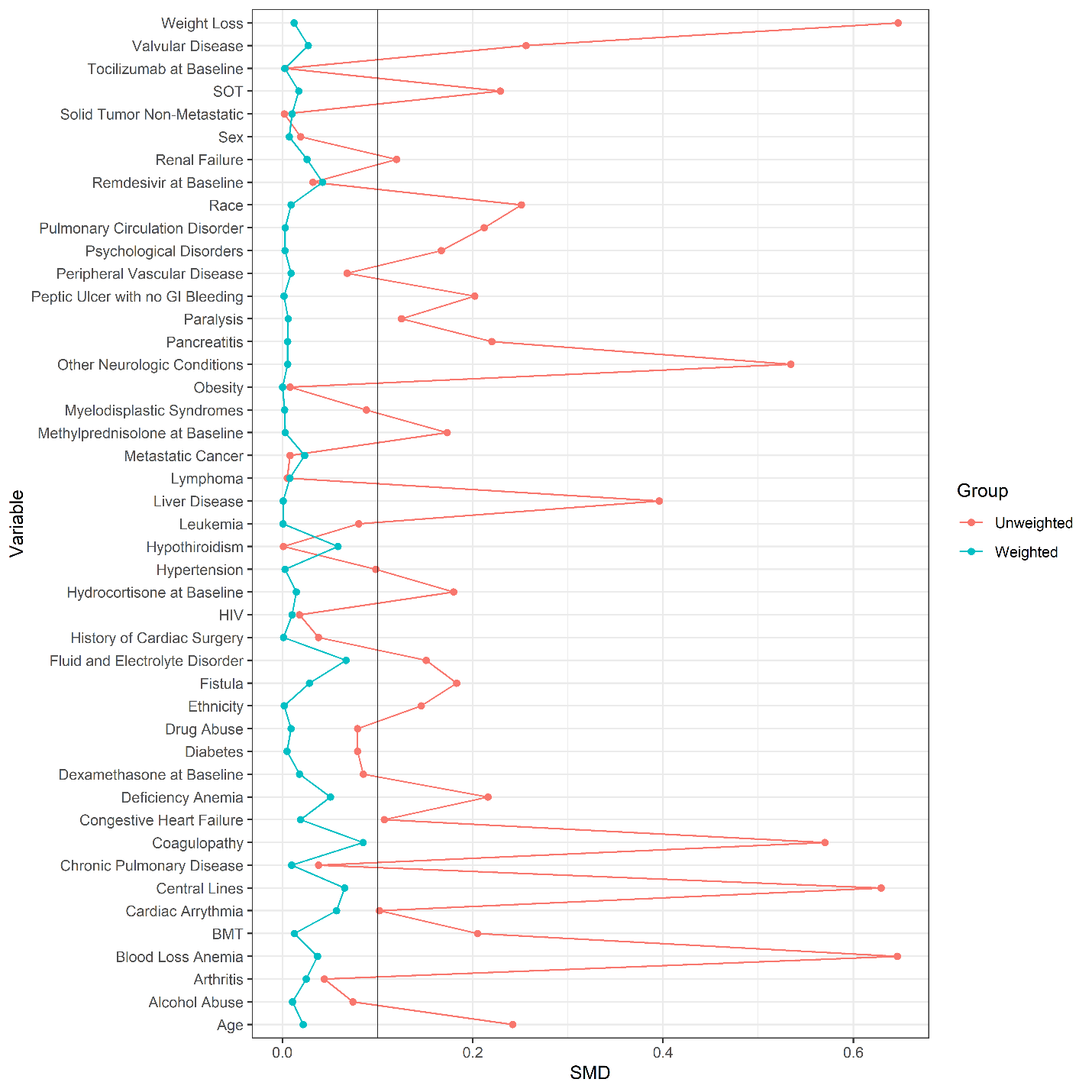


Appendix Figure 8. Incidence of COVID-19 associated invasive fungal infections among COVID-19 intubated patients


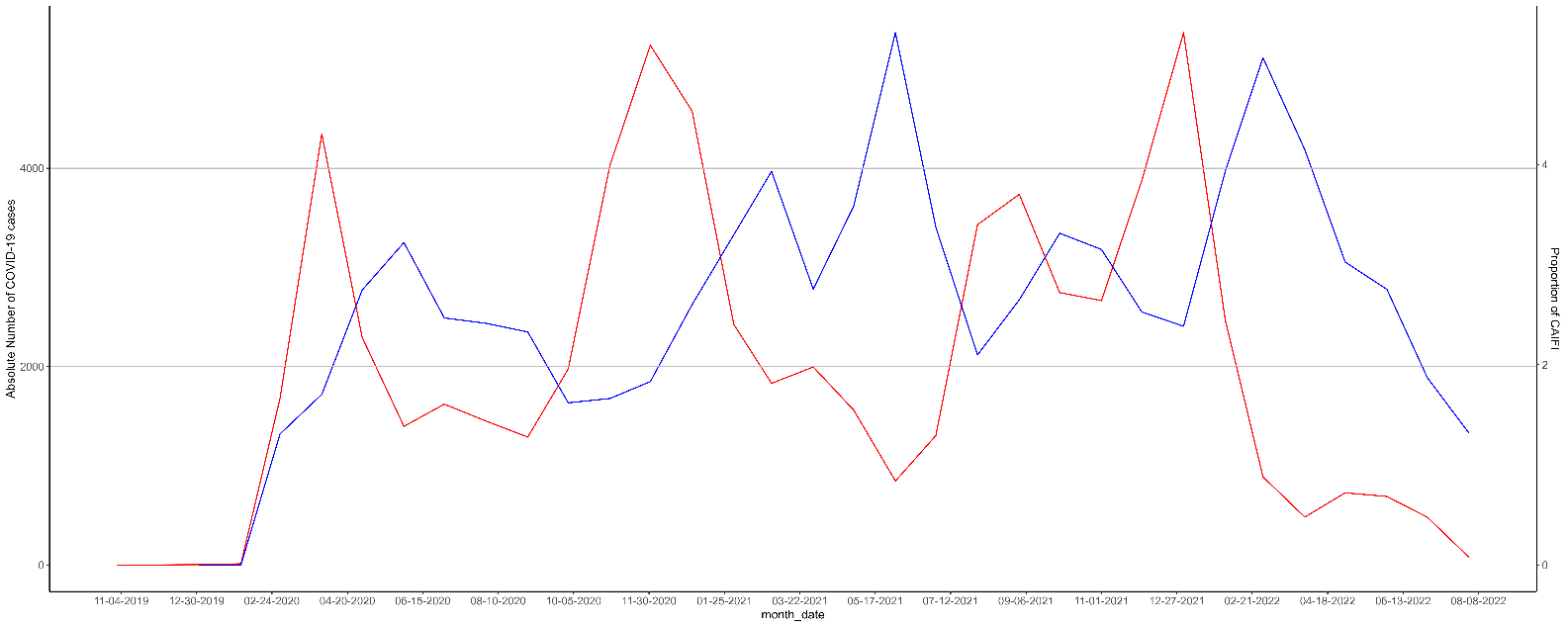


Red lines present the absolute number of COVID-19 cases in the left Y axis and blue lines represent proportion of CAIFI cases in the right Y axis.

Appendix Figure 9. Incidence of COVID-19 associated *Aspergillus* infections among COVID-19 intubated patients


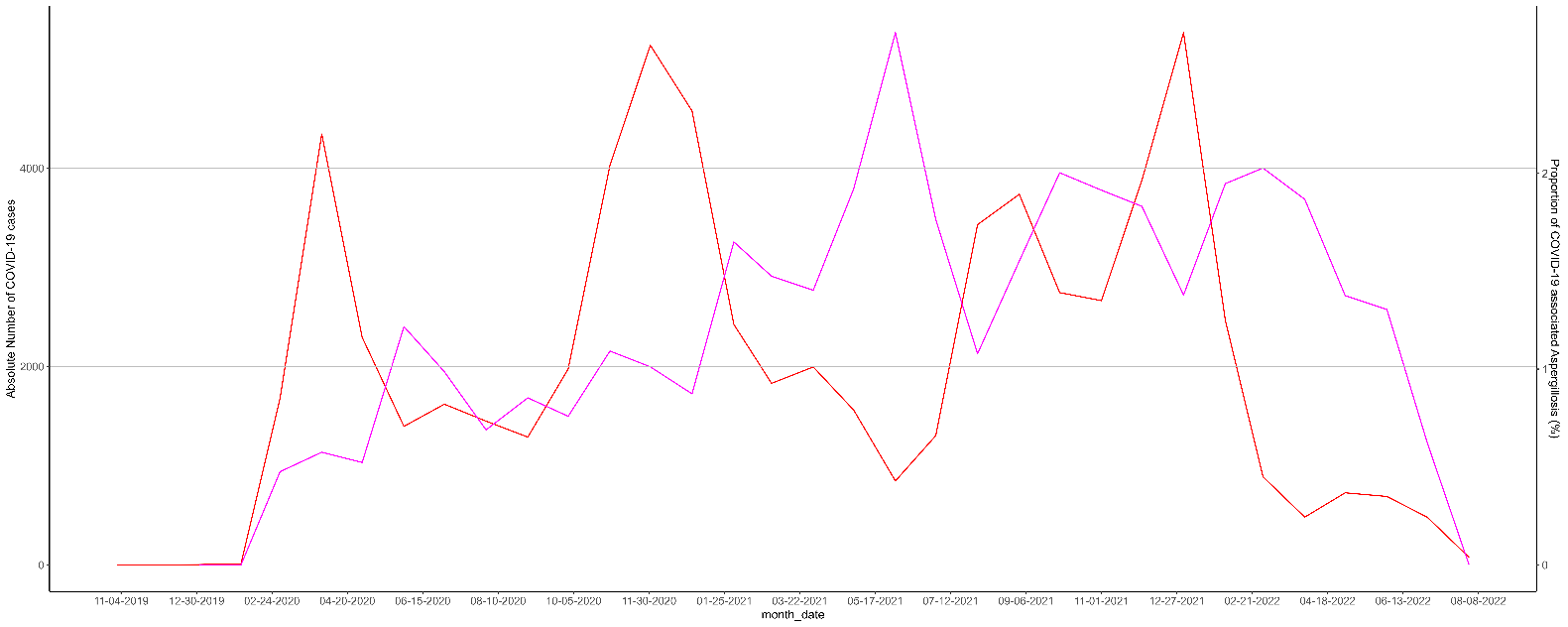


Red lines present the absolute number of COVID-19 cases in the left Y axis and magenta lines represent proportion of *Aspergillus* cases in the right Y axis.

Appendix Figure 10. Incidence of COVID-19 associated *Candida* infections among COVID-19 intubated patients


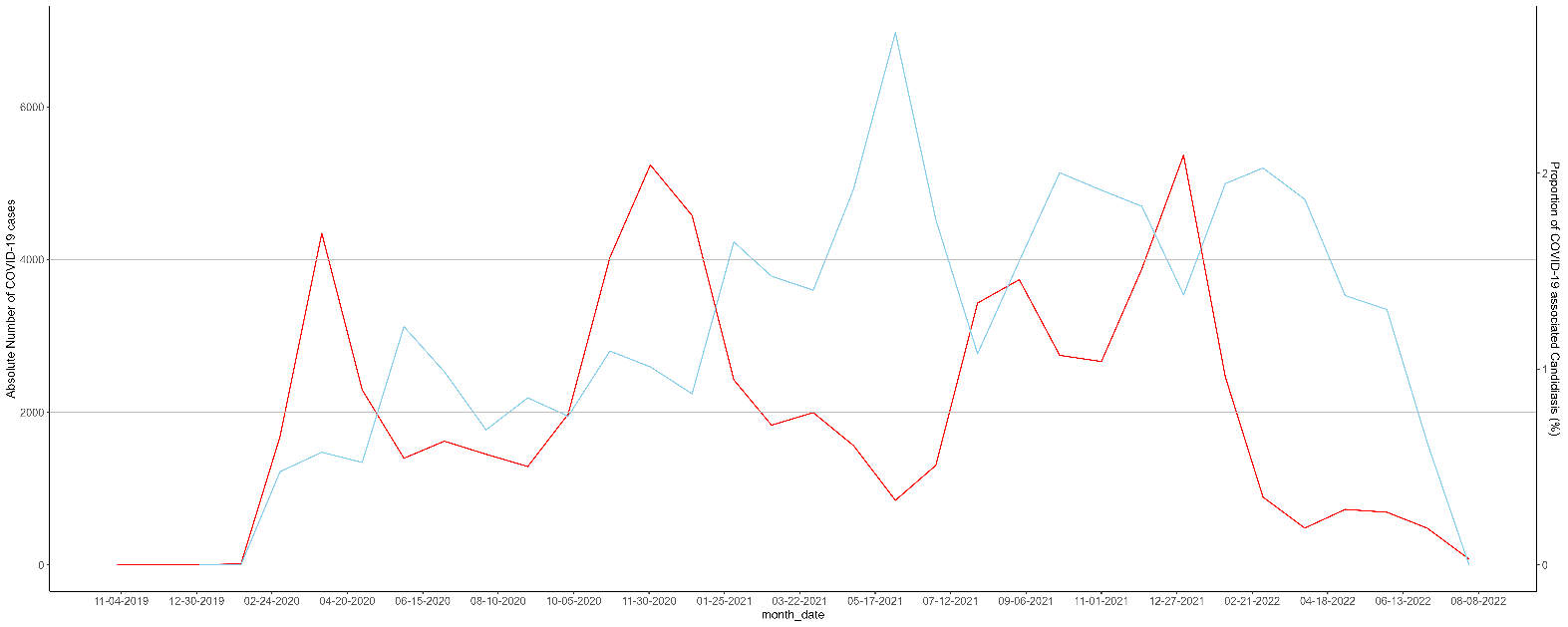


Red lines present the absolute number of COVID-19 cases in the left Y axis and light blue lines represent proportion of *Candida* cases in the right Y axis.

Appendix Figure 11. Incidence of COVID-19 associated *Cryptococcus* infections among COVID-19 intubated patients


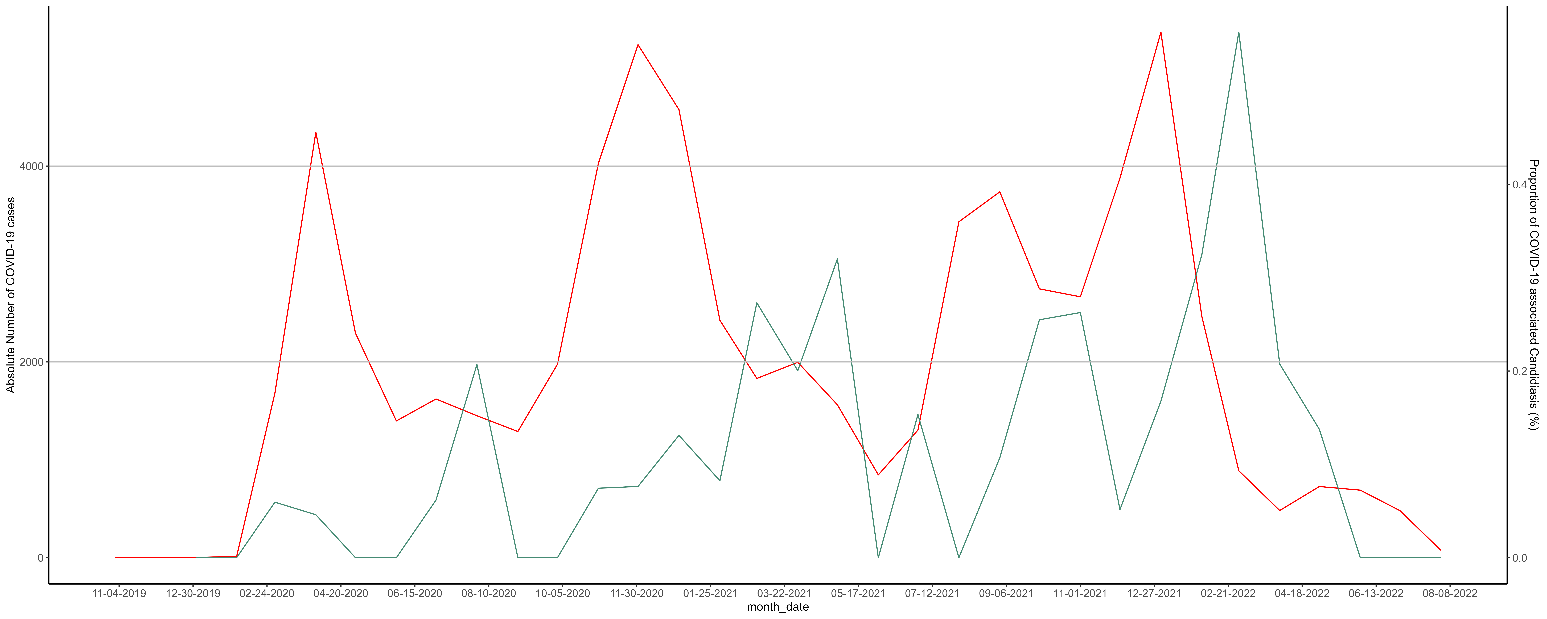


Red lines present the absolute number of COVID-19 cases in the left Y axis and green lines represent proportion of *Cryptococcus* cases in the right Y axis.

Appendix Figure 12. Incidence of COVID-19 associated *Histoplasma* infections among COVID-19 intubated patients


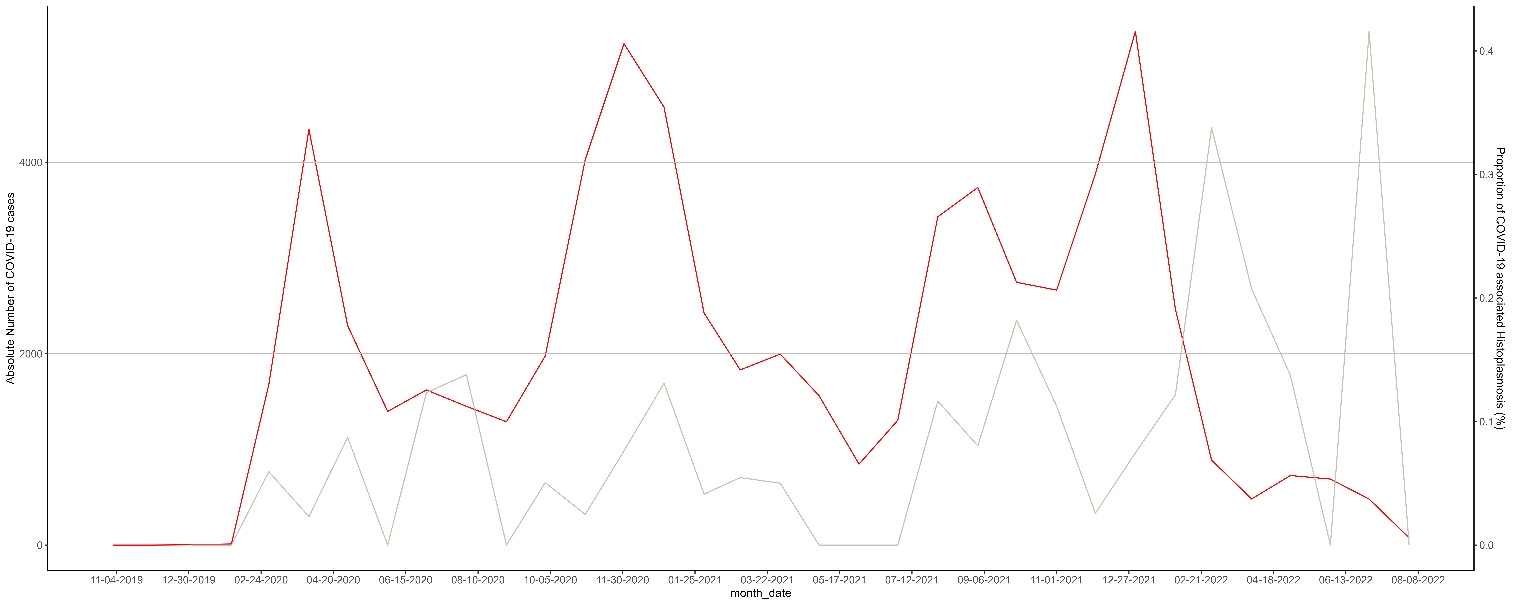


Red lines present the absolute number of COVID-19 cases in the left Y axis and brown lines represent proportion of *Histoplasma* cases in the right Y axis.

Appendix Figure 13. Incidence of COVID-19 associated *Blastomyces* infections among COVID-19 intubated patients


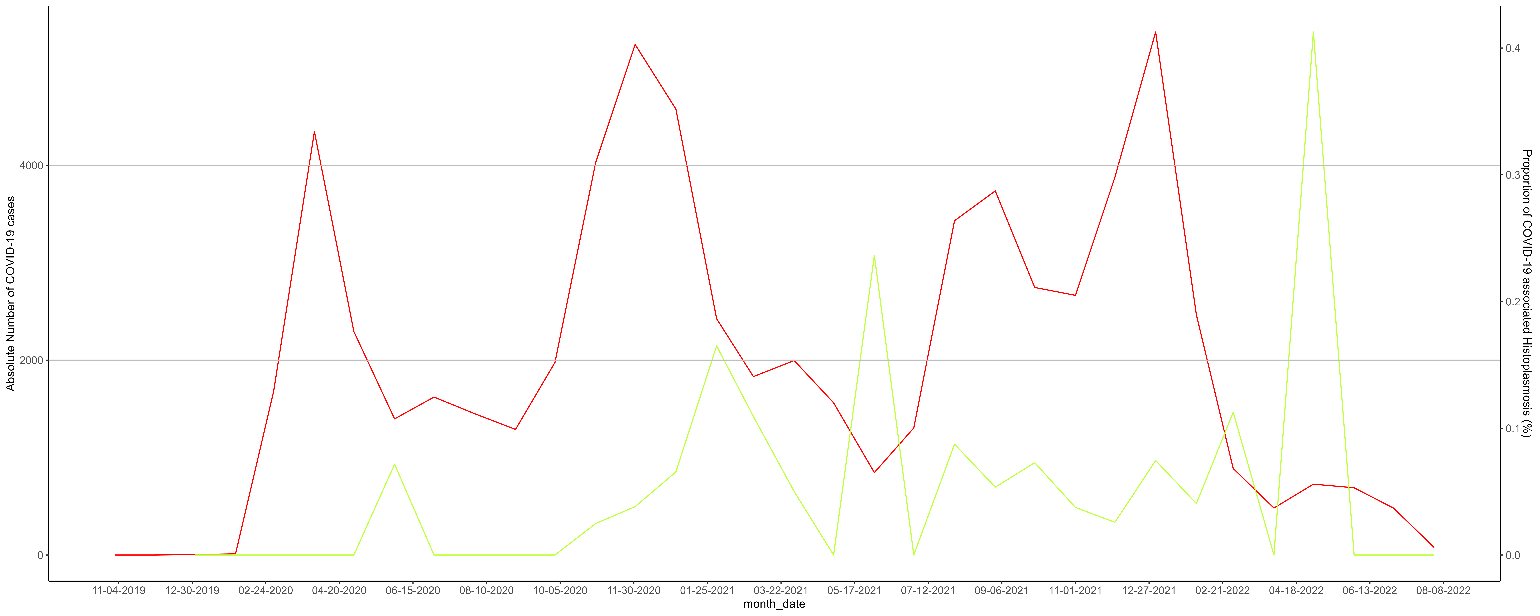


Red lines present the absolute number of COVID-19 cases in the left Y axis and yellow lines represent proportion of *Blastomyces* cases in the right Y axis.

Appendix Figure 14. Incidence of COVID-19 associated *Coccidioides* infections among COVID-19 intubated patients


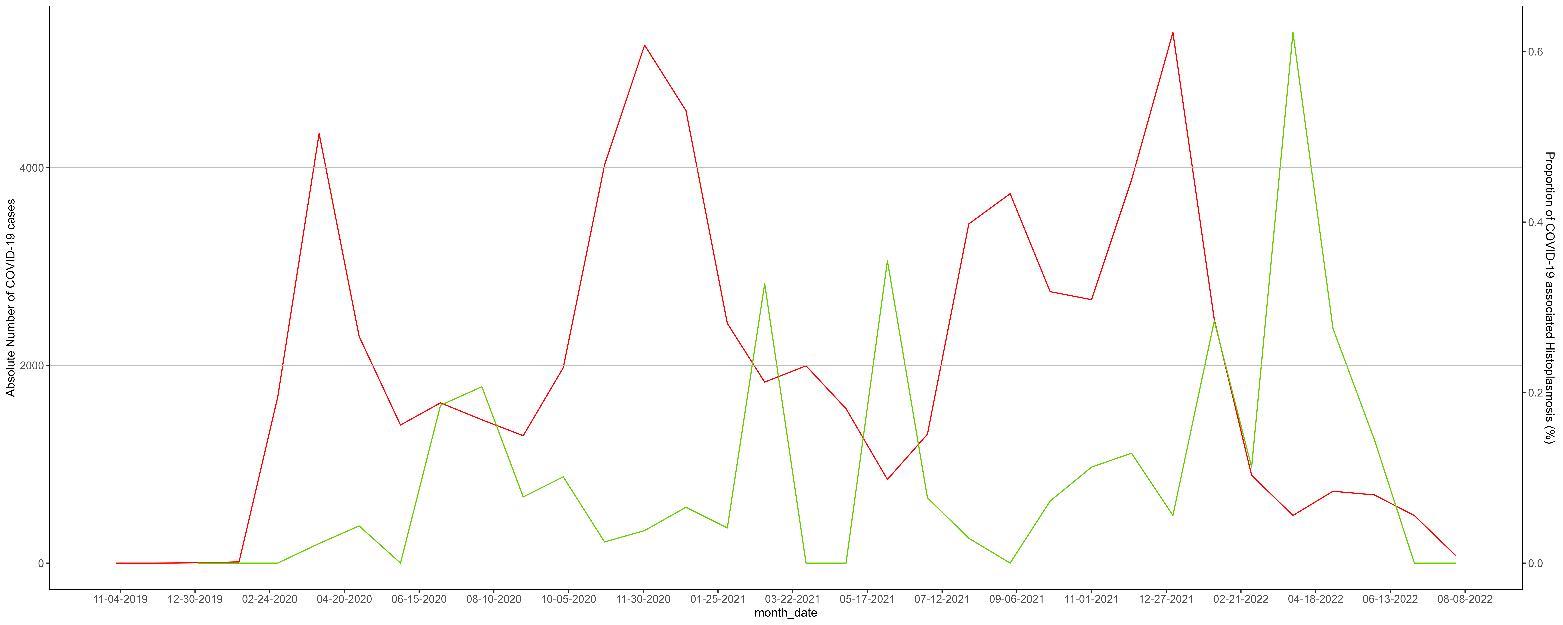


Red lines present the absolute number of COVID-19 cases in the left Y axis and light green lines represent proportion of *Coccidioides* cases in the right Y axis.

Appendix Figure 15. Incidence of COVID-19 associated Mucorales infections among COVID-19 intubated patients


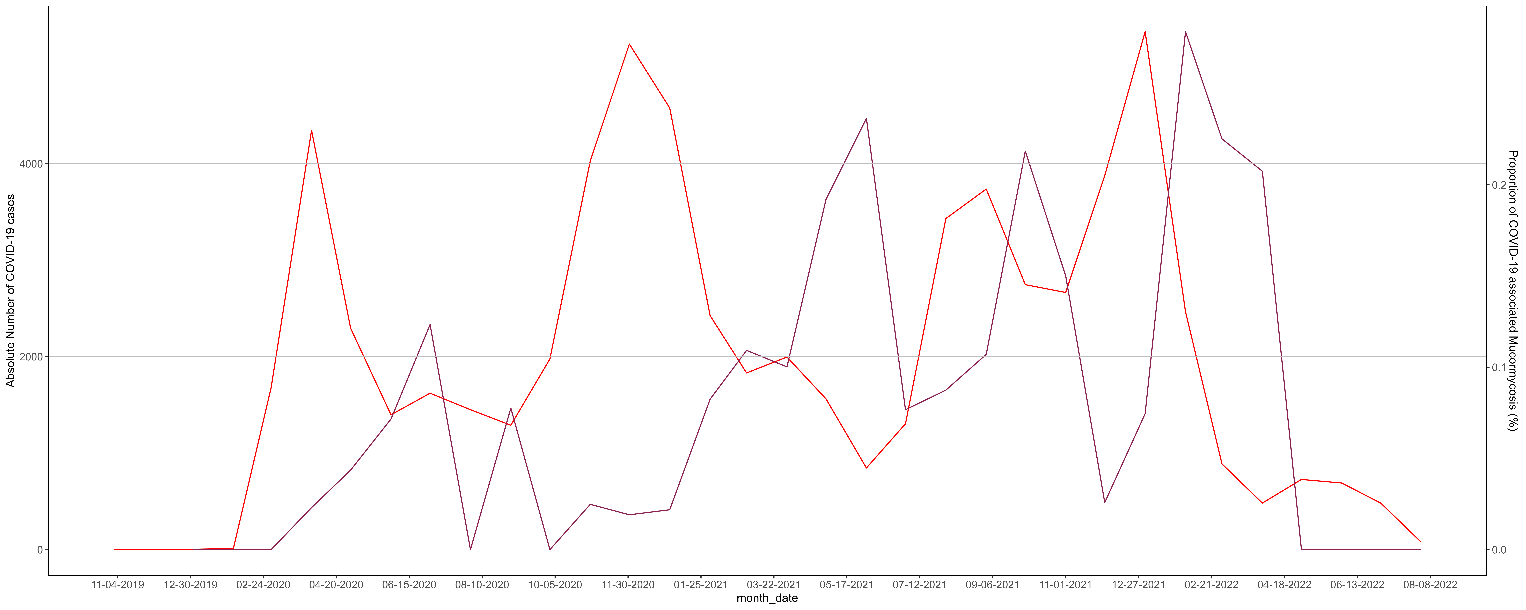


Red lines present the absolute number of COVID-19 cases in the left Y axis and purple lines represent proportion of Mucorales cases in the right Y axis.

Appendix Figure 16. Association between CAIFI and 30-day mortality among COVID-19 intubated patients by individual CAIFI


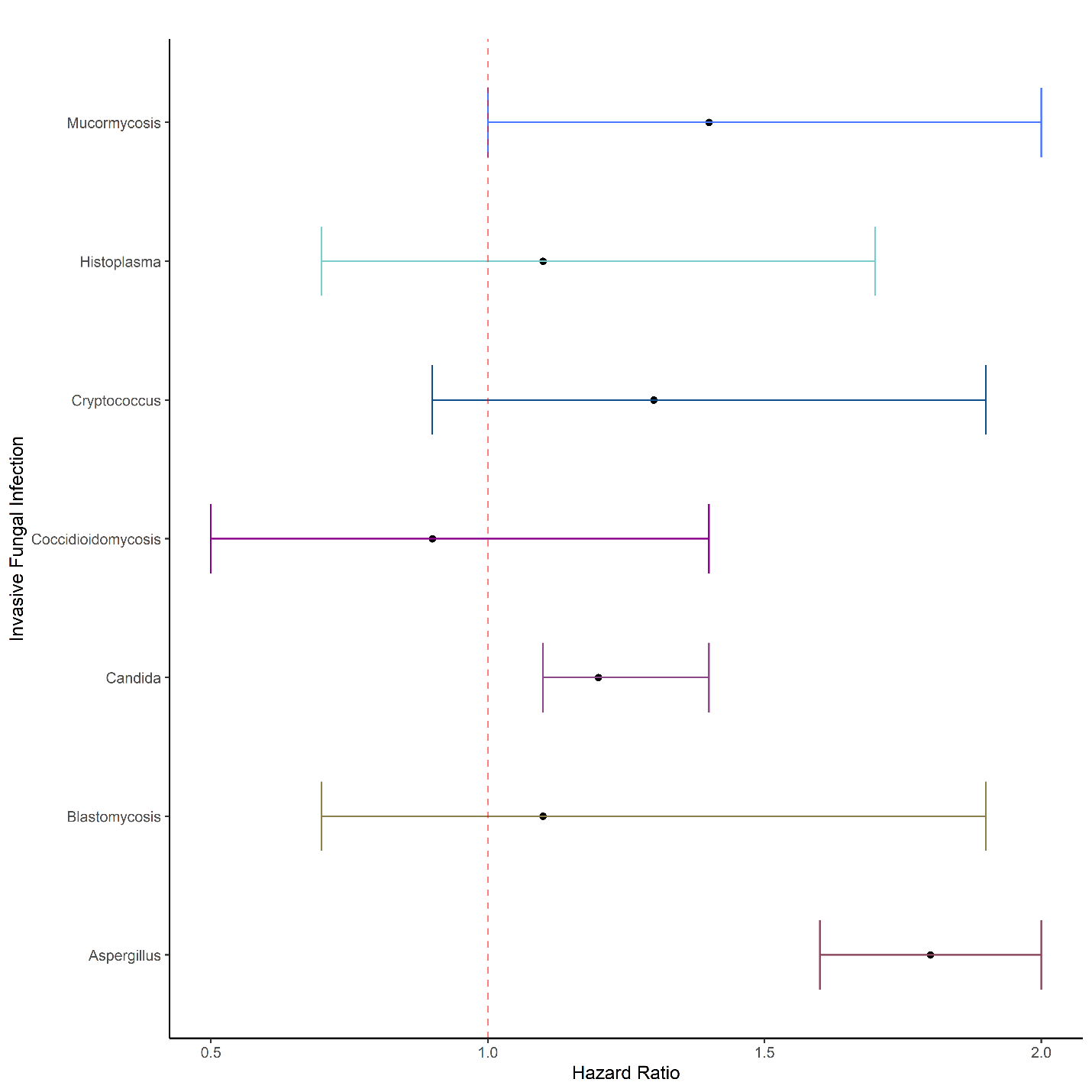


1. Xu S, Ross C, Raebel MA, Shetterly S, Blanchette C, Smith D. Use of stabilized inverse propensity scores as weights to directly estimate relative risk and its confidence intervals. Value Health. 2020;13(2):1-13.

2. Hernán M, Robbins J. Causal Inference: What If: Boca Raton: Chapman & Hall/CRC; 2020.

3. Austin PC. Balance diagnostics for comparing the distribution of baseline covariates between treatment groups in propensity-score matched samples. Statistics in Medicine. 2009;28:3083-107.

4. Spencer DJ. CDC Museum COVID-19 Timeline. Center for Diseases Control and Prevention; 2022 [09/25/22]; Available from: <https://www.cdc.gov/museum/timeline/covid19.html>.

5. Suissa S. Immortal Time Bias in Pharmacoepidemiology. American Journal of Epidemiology. 2008;167(4):492-9.

6. Funk MJ, Westreich D, Wiesen C, Sturmer T, Brookhart MA, Davidian M. Doubly Robust Estimation of Causal Effects. American Journal of Epidemiology. 2011;137(7):761-7.

7. Wheat LJ, Freifeld AG, Kleiman MB, Baddley JW, McKinsey DS, Loyd JE, et al. Clinical practice guidelines for the management of patients with histoplasmosis: 2007 update by the Infectious Diseases Society of America. Clin Infect Dis. 2007 Oct 1;45(7):807-25.

8. Thompson GR, 3rd, Le T, Chindamporn A, Kauffman CA, Alastruey-Izquierdo A, Ampel NM, et al. Global guideline for the diagnosis and management of the endemic mycoses: an initiative of the European Confederation of Medical Mycology in cooperation with the International Society for Human and Animal Mycology. Lancet Infect Dis. 2021 Dec;21(12):e364-e74.

9. Perfect JR, Dismukes WE, Dromer F, Goldman DL, Graybill JR, Hamill RJ, et al. Clinical practice guidelines for the management of cryptococcal disease: 2010 update by the infectious diseases society of america. Clin Infect Dis. 2010 Feb 1;50(3):291-322.

10. Pappas PG, Kauffman CA, Andes DR, Clancy CJ, Marr KA, Ostrosky-Zeichner L, et al. Clinical Practice Guideline for the Management of Candidiasis: 2016 Update by the Infectious Diseases Society of America. Clin Infect Dis. 2016 Feb 15;62(4):e1-50.

11. Cornely OA, Alastruey-Izquierdo A, Arenz D, Chen SCA, Dannaoui E, Hochhegger B, et al. Global guideline for the diagnosis and management of mucormycosis: an initiative of the European Confederation of Medical Mycology in cooperation with the Mycoses Study Group Education and Research Consortium. Lancet Infect Dis. 2019 Dec;19(12):e405-e21.
